# Electrospinning for Mimicking Bioelectric Microenvironment in Tissue Regeneration

**DOI:** 10.34133/research.0959

**Published:** 2025-11-10

**Authors:** Zhuowen Hao, Zepu Wang, Ying Wang, Minchao Dong, Zheyuan Zhang, Jiayao Chen, Guang Shi, Junwu Wang, Renxin Chen, Zouwei Li, Xin Zhao, Jingfeng Li

**Affiliations:** ^1^ Department of Orthopedics, Zhongnan Hospital of Wuhan University, Wuhan 430071, China.; ^2^ Department of Applied Biology and Chemical Technology, The Hong Kong Polytechnic University, Hong Kong SAR, China.; ^3^ Department of Obstetrics and Gynecology, Renmin Hospital of Wuhan University, Wuhan 430060, China.; ^4^ The Hong Kong Polytechnic University Shenzhen Research Institute, Shenzhen 518057, China.

## Abstract

Electric signals exert critical roles in tissue regeneration. Electrotherapeutic devices in the clinic have confirmed clinical effectiveness, but they may cause low patient compliance and infection risks due to invasive electrodes and external power sources. Through electrospinning, electroactive electrospun scaffolds address these shortcomings. This review first outlines the physiological cues of electric signals in electrosensitive tissue regeneration and signaling pathways induced by electric stimulations for tissue regeneration. Next, it details basic fabrications for extracellular matrix mimetic scaffolds, emphasizing the endowment of surface potential by voltage polarity and the selection of electrospinning methods and materials. Then, it critically analyzes methodologies to imbue scaffolds with electroconductivity to facilitate cell-to-cell signaling and piezoelectric effects or triboelectrification to form electrical cues for tissue regeneration. Moreover, smart applications of electroactive electrospun scaffolds for mimicking bioelectric niches are summarized, including conductive or piezoelectric electrospun scaffolds, electroactive composite implants, self-powered nanogenerators, and smart electroactive drug delivery devices. Finally, current challenges and future directions toward clinical implementation are discussed.

## Introduction

Tissue defects generally result from injuries, illnesses, surgical removal, or congenital problems, necessitating the regeneration of defective tissues and the restoration of organ functions [1]. For a long time, autografts or allografts are mainly used in the repair or regeneration of defective defects. However, autografts are correlated with additional surgery, limited availability, and potential adverse effects at the donor site, including infection, persistent pain, and hemorrhage, and allografts may cause infection transmission and immune-mediated rejection of transplanted tissue [2,3]. Despite these, tissue and organ transplantation are still the most common methods to treat tissue defects, but the number of grafts fail to meet the urgent clinical needs due to the dramatic increase of the transplant waiting list, which causes a decrease in the patient’s life quality and even results in the patient’s death [4,5]. Thus, there has been a substantial push and exploration to develop strategies for creating replacement tissues that mimic autografts.

Electrospinning is a promising technique to fabricate fiber-based scaffolds that simulate natural extracellular matrix (ECM) in various tissues such as skin [6], liver [7], and spinal cord [8] because electrospun scaffolds show unique properties including large surface area, microscale fiber structure, tailored surface nanotopography, and controlled mechanical properties [9,10]. The orientation of electrospun fibers can be easily controlled to mimic the aligned structures such as skeletal muscle [11], tendon fascicles [12], and peripheral nerve [13]. With the advancement of electrospinning technology, 3-dimensional (3D) electrospun structures can be also fabricated to simulate complex tissue architecture [14]. Therefore, electrospun scaffolds have been used to some extent in different tissue regeneration applications, but the suboptimal function and the limited efficacy of electrospun scaffolds alone cannot meet the multiple requirements of tissue regeneration. To improve the regeneration efficiencies, bioactive factors (e.g., bioactive peptides/proteins [15], functional nanoparticles [16], and nucleic acids [17]) or biophysical stimuli (e.g., electricity [18], magnetic field [19], and photothermal effect [20]) are finely integrated with electrospun scaffolds to promote tissue regeneration. Compared to bioactive factor delivery, integrating biophysical stimuli avoids high loading doses and burst release of bioactive factors while providing precise manipulation and sustained repair effects, showing promising potential for tissue regeneration [21,22].

Bioelectricity is a fundamental biophysical factor in human physiology and tissue regeneration because it exerts potent roles in mediating tissue function and controlling cell behavior in electroactive tissues such as nerve system, heart, skin, muscle, and bone [23]. Currently, electrotherapeutic devices for bone, muscle, nerve, and brain are generally used in clinics to promote tissue regeneration and repair, but they are correlated with invasive electrode and external power supply, causing low patient compliance and infection risk [24]. Therefore, electroactive scaffolds have been developed for tissue regeneration, and there are mainly 2 strategies. One strategy is to impart charges on the scaffold surface because surface charges can modulate cell adhesion, proliferation, and fate direction [25,26]. In particular, negatively charged surfaces help promote osteogenic differentiation and mineralization, while positively charged surfaces facilitate protein adsorption, initial cell adhesion and spreading, and immunomodulation, and exert certain antibacterial effects [27,28]. The other strategy is to directly use electroactive materials to fabricate scaffolds to achieve electroconductivity to facilitate intercellular electrical signal transmission or realize self-powered properties to provide electrical signals on the basis of distinctive mechanisms such as piezoelectric effects and triboelectrification [29,30]. Therefore, both strategies, regulating cell behavior via surface potential or using electroactive materials to achieve conductivity or self-powering properties, can effectively advance the effective application of electric stimulations in tissue regeneration.

**Fig. 1.**
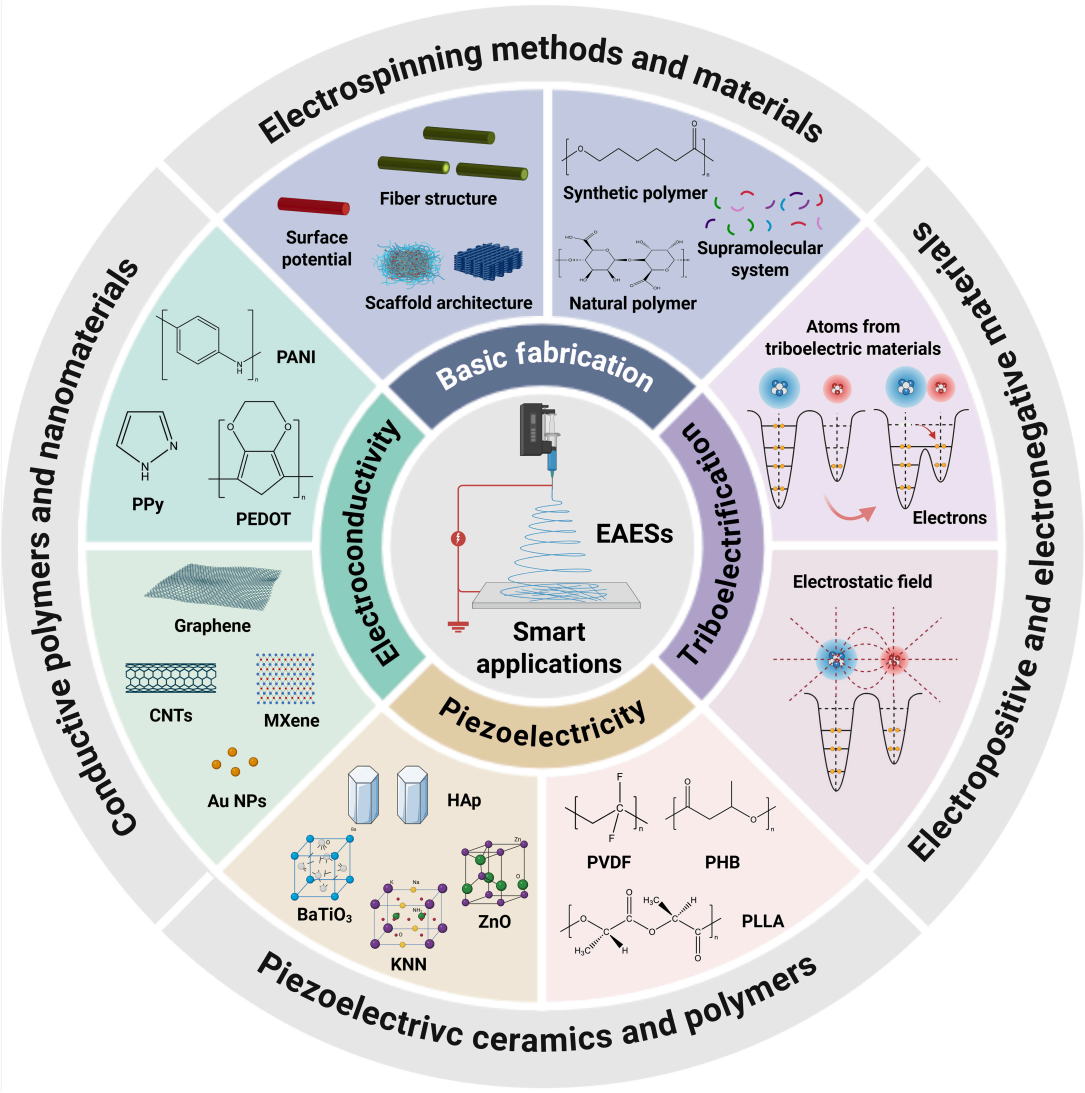
Scheme of electrospinning for mimicking bioelectric niches for tissue regeneration, including basic fabrication by electrospinning methods and materials, electroconductivity by conductive polymers and nanomaterials, piezoelectricity by piezoelectric ceramics and polymers, and triboelectrification by electropositive and electronegative materials.

Electrospinning is a potent and promising technology for fabricating electroactive electrospun scaffolds (EAESs), as it can not only endow the scaffolds with long-term and stable surface potential by controlling voltage polarity to regulate the molecular orientation within fibers, but also directly utilize electroactive materials to construct conductive or self-powered scaffolds. Although some studies focused on electrospinning in tissue engineering and regenerative medicine [31–33], a comprehensive review of electrospinning to fabricate EAESs to simulate bioelectric microenvironment to promote tissue regeneration remains lacking. Herein, this review helps provide systematic strategies to simulate ECM characteristics and electroactivity by fabricating EAESs and presenting their smart application in tissue engineering (Fig. 1). It first focuses on physiological cues of electric signals and distinctive signaling pathways to induce electrosensitive tissue regeneration. The basic fabrications are comprehensively summarized corresponding to the endowment of surface potential by voltage polarity and the selection of electrospinning methods and materials. Then, this review focuses on how to endow electrospun scaffolds with electroconductivity, piezoelectricity, or triboelectrification to induce tissue regeneration. Moreover, smart applications of EAESs for mimicking bioelectric niches are summarized, including conductive or piezoelectric electrospun scaffolds, electroactive composite implants, self-powered nanogenerators, and smart electroactive drug delivery devices. Finally, the review addresses current obstacles and explores forthcoming research directions, aiming to promote the clinic translation and application of EAESs in the clinic.

## Physiological Cues of Bioelectric Stimulations to Induce Tissue Regeneration

**Fig. 2.**
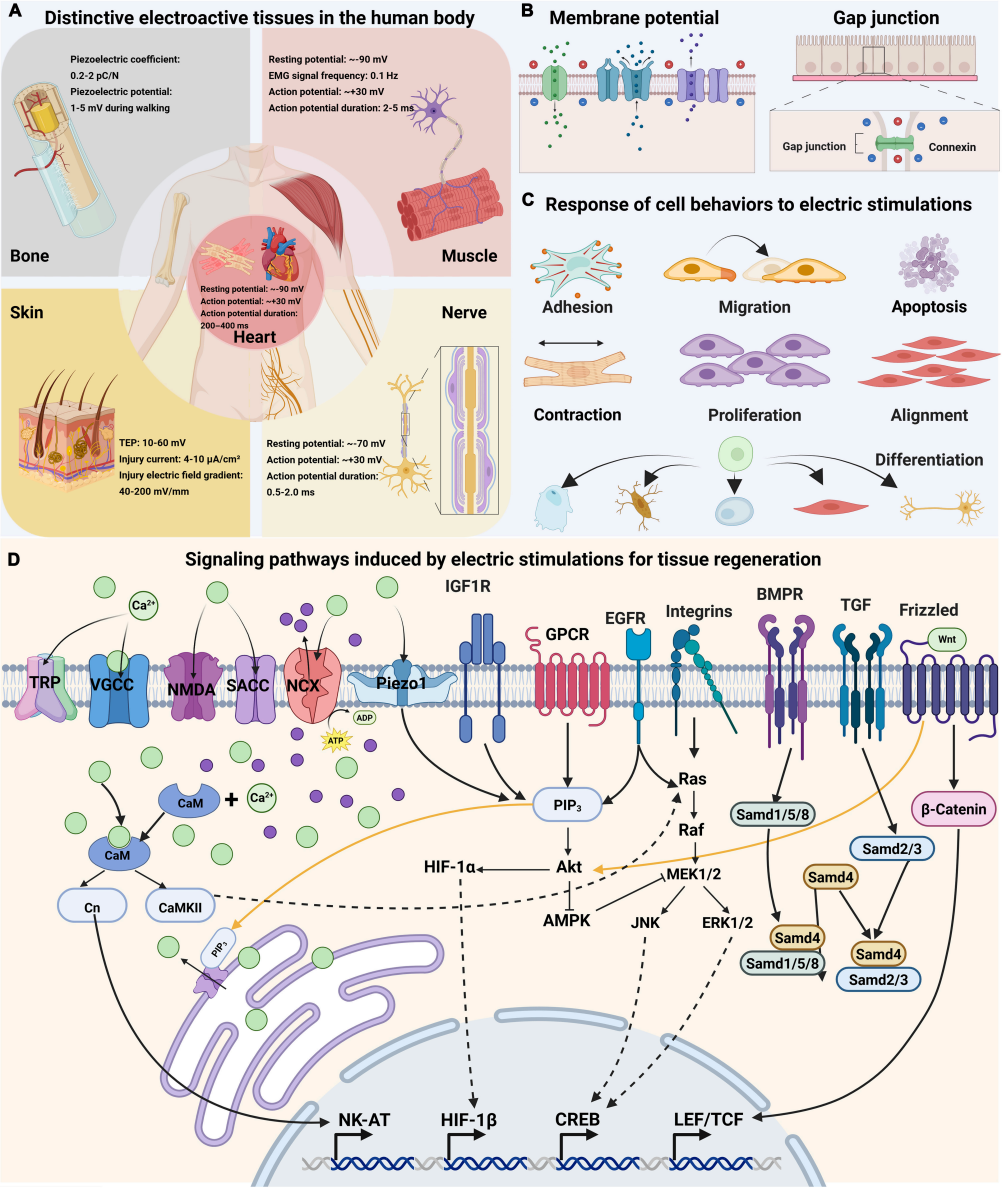
Physiological cues of electric signals to induce tissue regeneration. (A) Distinctive electroactive tissues in the human such as bone, muscle, skin, nerve and heart. (B) Cell-level mechanisms induced by electric stimulations by membrane potential and gap junction. (C) Response of cell behaviors to electric stimulations. (D) Signaling pathways induced by electric stimulations for tissue regeneration.

Bioelectricity is fundamental to essential physiological processes in the human body. There are various electroactive tissues including bone, muscle, skin, nerve, and heat, and bioelectricity provides the essential electrophysiological basis for their functions and regeneration (Fig. 2A). At the cell level, virtually all human cells maintain a long-term, steady-state membrane potential (*V*_m_), which ranges from −10 to −90 mV depending on the cell type. Membrane potential serves as a key indicator of cellular responsiveness to electric stimulations, and the membrane potential can also be influenced by adjacent cells via gap junction (Fig. 2B). The change of membrane potential could further modulate cell behaviors including adhesion, contraction, migration, proliferation, alignment, differentiation, and apoptosis (Fig. 2C). Herein, this section mainly focuses on the electric signals in distinctive electrosensitive tissues and mechanisms of cell response induced by electric stimulations.

### Electric signals in distinctive electrosensitive tissues

Electric signals are one of the driving forces of tissue regeneration, and this process centers on the formation of endogenous electric fields in conjunction with cellular electrical response mechanisms [34]. This regulatory mechanism plays a central role in the physiological maintenance and development of electroactive tissues including nerve, heart, bone, skin, and skeleton muscle. In the nervous system, action potentials are the foundation of information transmission. The directional conduction of nerve impulses through transmembrane potential oscillations mediated by ion channels not only integrates sensory, motor, and cognitive functions in real time, but also guides the extension of axons in a specific direction and the formation of synapses during neural development. Endogenous electric fields promote the differentiation of neural stem cells into neurons and inhibit the over-proliferation of glial cells by regulating calcium ion inward flow and other pathways. This provides a molecular basis for repairing nerve injuries [35]. The heart’s electrical activity exhibits a high degree of coordination. Rhythmic electrical impulses are generated spontaneously by the sinus node and conducted synchronously to the ventricular myocardium through the His-Purkinje system. This forms precise excitation–contraction coupling. This network ensures that the atria and ventricles contract sequentially to maintain effective cardiac output [36]. The electrical activity of bone tissue stems from its unique piezoelectric properties. Under mechanical stress, the noncentrosymmetric structure of collagen fibers becomes polarized, converting mechanical stimuli into local electric fields that activate signaling in the osteoblast network [37]. The electrical signaling system of skin tissue exhibits a stable transepithelial potential (TEP), and epithelial skin cells have potential differences of 10 to 60 mV, which create intracellular negative electrical microenvironments [38]. When tissue is injured, short-circuiting of the TEP leads to a lateral electric field gradient of 40 to 200 mV/mm between the site of the injury and the surrounding tissue to drive the directional flow of Cl^-−^, Na^+^, and other ions, creating an endogenous current of 4 to 10 μA/cm^2^ [39]. This physiological process activates an electrochemical effect. Keratinocytes migrate toward the cathode in a field of 10 to 400 mV/mm, while fibroblasts and endothelial cells migrate toward the anode in a field of greater than 200 mV/mm [39]. Electrical signaling in skeletal muscle is characterized by classical excitation–contraction coupling, and the release of acetylcholine from motor neurons triggers the depolarization of the muscle membrane [40]. Then, action potentials travel along the T-tubules to the sarcoplasmic reticulum to trigger calcium release and myofilament gliding, and the strength of muscle contraction is directly determined by the efficiency of this electromechanical conversion process [41]. Therefore, electric signals form a multilevel regulatory network in 5 major physiological systems: the nervous system encodes and transduces information, the heart ensures rhythmic pumping, the skeleton converts mechanical stimuli into biochemical signals, the skin coordinates the injury response, and skeletal muscle performs electromechanical conversion. These systems share core mechanisms, including ion channel regulation, stem cell-directed differentiation, and regenerative microenvironment construction, which provide a bionic foundation for tissue engineering.

### Common signaling pathways induced by electric stimulations

Endogenous or exogenous electric stimulations promote tissue regeneration by affecting cell membrane potential as well as the membrane potential of surrounding cells by gap junctions, and these regulations occur mainly through ion channels, signaling pathways, and transcriptional regulation. Examples of these pathways include Ca^2+^ signaling pathway, mitogen-activated protein kinase (MAPK) signaling pathway, phosphatidylinositol 3-kinase (PI3K)/protein kinase B (Akt) signaling pathway, and Wnt signaling pathway, as well as growth factor-related signaling pathway (Fig. 2D).

#### Ca^2+^ signaling pathway

Distinctive ion channels on the cell membrane are correlated with the flow of Ca^2+^ from the extracellular space into the cell, including activating voltage-gated calcium channels, transient receptor potential channels, stretch-activated calcium channels, and the Na^+^–Ca^2+^ exchanger [42]. Ca^2+^ can also be released from the endoplasmic reticulum by the IP₃ pathway, which is correlated with G protein-coupled receptor signaling [43]. In general, electric stimulations mainly act on voltage-gated calcium channels to mediate Ca^2+^ influx [44]. Ca^2+^ binds to calmodulin (CaM) to form a Ca^2+^/CaM complex, which activates CaM-dependent kinase (CaMK) and calcineurin (CaN), thereby stimulating transcriptional regulation [45]. Moreover, the Ca^2+^ influx may be the initial signaling of electric stimulations to induce other downstream signaling pathways, and the Ca^2+^ signaling pathway has shown critical effects of electric stimulations for multiple tissue regeneration such as nerve [46], bone [47], and skin [48].

#### MAPK signaling pathway

The MAPK signaling pathway plays a crucial role in cellular responses to external stimuli, and it primarily consists of 3 classical subfamilies: extracellular signal-regulated kinases (ERK), c-Jun N-terminal kinases (JNK), and p38 MAPK, each of which contributes distinctly to various biological functions and pathological processes [49]. For the effects of electric stimulations on tissue regeneration, the MAPK/ERK signaling pathway and the p38 MAPK signaling pathway mainly involve distinctive tissues [50]. For example, the MAPK/ERK signaling pathway has been reported to participate in the neurogenic differentiation of neural stem cells for the recovery of brain tissues [51]. The ERK-dependent pathway is also involved in the angiogenesis of endothelial cells and the migration of lens epithelium [52,53]. On the other hand, the activation of p38 MAPK signaling has been reported to be correlated with osteogenic differentiation for bone regeneration and chondrogenic differentiation for cartilage healing [54]. Therefore, the MAPK signaling pathway plays an important role in electric stimulations for tissue regeneration, and different sub-signaling may be activated in different repair cells.

#### PI3K/Akt signaling pathway

The PI3K/Akt signaling pathway plays a pivotal role in critical cellular processes including survival, proliferation, differentiation, and metabolism [55,56]. Upon extracellular stimulation, PI3K becomes activated and catalyzes the conversion of phosphatidylinositol 4,5-bisphosphate (PIP_2_) to phosphatidylinositol 3,4,5-trisphosphate (PIP_3_) at the plasma membrane [55]. Serving as a second messenger, PIP_3_ recruits and activates Akt, and then activated Akt phosphorylates a wide range of downstream target proteins, thereby ultimately regulating multiple biological processes [55]. The PI3K/Akt signaling pathway has been shown to be closely associated with electrical stimulation-mediated tissue regeneration, and this relationship has been demonstrated in a variety of tissues such as bone [57], skin [58], and nerve [59]. The activation of the PI3K/Akt signaling pathway may be correlated with initially increased Ca^2+^ concentration under electric stimulation [60]. In addition, signaling is also involved in immune regulation such as M2 macrophage polarization and angiogenesis by the secretion of growth factors for tissue regeneration [61,62]. Therefore, the PI3K/Akt signaling pathway activated by electrical stimulation not only directly participates in the growth and differentiation of repair cells but also modulates the tissue microenvironment to promote regeneration.

#### Wnt signaling pathway

The Wnt signaling pathway is a signaling cascade initiated by the binding of Wnt ligand proteins to membrane receptor complexes, leading to the activation of multiple downstream branches [63]. Based on its dependence on β-catenin-mediated transcriptional activation, the pathway is broadly categorized into the canonical pathway (i.e., the Wnt/β-catenin signaling pathway) and noncanonical pathways, which include the Wnt/planar cell polarity pathway and the Wnt/Ca^2+^ pathway [63]. Both the canonical Wnt/β-catenin pathway and the noncanonical Wnt/Ca^2+^ pathway has been reported to be associated with electrical stimulation-mediated bone regeneration [64]. In another study, the Wnt/β-catenin signaling pathway was activated by produced miRNA form Schwann cells (SCs) that were subjected to the electric stimulation for bone regeneration [65]. In addition to bone regeneration, Wnt signaling is also activated by electric stimulations in the regeneration of other tissues such as nerve [66], spinal cord [67], cartilage [68], and hair follicle [69].

#### Growth factor-related signaling pathway

Electrical stimulation can induce stem cells or tissue-specific reparative cells to secrete various growth factors, which subsequently activate their corresponding signaling pathways to facilitate tissue repair. For example, electric stimulation could increase the endogenous production of transforming growth factor beta 1 (TGF-β1), and then secreted TGF-β1 could promote the osteogenesis by inducing the TGF-β/Smad2 signaling pathway [70]. In another study, electric stimulation could induce the secretion of FGF2 from fibroblasts, and then FGF2 could activate the MAPK/ERK signaling pathway and promote the production of vascular endothelial growth factor (VEGF) by human umbilical vein endothelial cells (HUVECs), showing great potential for wound healing [71]. In addition, electric stimulation could mediate mesenchymal stem cells (MSCs) to secrete VEGF and hepatocyte growth factor for ischemic tissue regeneration [72].

### Signaling pathways for stem cell differentiation induced by electric stimulations

Stem cells, as the main repair cells, can proliferate and differentiate into specific functional cells to repair and regenerate damaged tissues [73]. Distinctive electric stimulations can modulate the behaviors and functions of stem cells, such as orientation, elongation, migration, and genome transcription [74–76]. More importantly, electric stimulations exert critical roles in determining stem cell fate or guiding stem cell differentiation into specific cell phenotypes (such as osteogenic, chondrogenic, neurogenic, cardiomyogenic, and angiogenic) for tissue regeneration (Table 1).

**Table 1.** Signaling pathways for stem cell differentiation induced by electric stimulations

Fate directions	Target tissues	Stem cell types	Types of electric stimulations	Electric stimulation parameters	Signaling pathways	Cell behaviors and responses	Ref.
Osteogenic	Bone	Bone marrow mesenchymal stem cells	Piezoelectric effects	Output voltage (800 mV), output current (0.4 μA)	Ca^2+^ signaling pathway, calcinuerin/CAMKII/NFAT signaling pathway	Activated voltage-gated calcium channels, osteogenic differentiation, improved expressions of BMP2, ALP, Runx2, OCN, OPN, and Col-I	[77]
	Bone	Mesenchymal stem cells	Direct current electric fields	Electric field strength (200 mV/cm)	Ca^2+^ signaling pathway, Ca^2+^/calcinuerin/CAMKII/NFAT signaling pathway	The formation of plasma membrane protrusions, the transport of Connexin 43 to membrane protrusions, activated voltage-gated calcium channels, osteogenic differentiation	[45]
	Bone	Bone marrow mesenchymal stem cells	Direct currents	Output current (4 μA)	Ca^2+^ signaling pathway, BMP2/Smad 5 signaling pathway	Activated voltage-gated calcium channels, adenosine triphosphate-actin remodeling, osteogenic differentiation, improved expressions of BMP2 and OCN	[78]
	Bone	Bone marrow mesenchymal stem cells	Currents with biphasic electric pulses	Output voltage (35 V), output current (3.7 μA)	Ca^2+^ signaling pathway, PI3K/AKT signaling pathway, WNT signaling pathway and MAPK signaling pathway	Activated voltage-gated calcium channels, improved expressions of PIEZO1 and PIEZO 2, osteogenic expressions, improved expression of OCN	[79]
	Bone	Mesenchymal stem cells	Piezoelectric effects	Output voltage (190 mV), piezoelectric coefficient d_14_ (1.82 pm/V)	Ca^2+^ signaling pathway, PI3K/AKT signaling pathway	Activated voltage-gated calcium channels, osteogenic differentiation, improved expressions of BMP2, OCN, Runx2, and Col-I	[80]
	Bone	Bone marrow mesenchymal stem cells	Surface negative charge	Surface static voltage (−0.51 kV), zeta potential (−57.3 mV)	FAK/ERK signaling pathway	Improved expressions of integrin α1, α2, α5, and β, osteogenic differentiation, improved expressions of ALP, OPN, OCN, and Col-I	[81]
	Bone	Bone marrow mesenchymal stem cells	Surface negative charge; electric fields with biphasic pulses	Surface potential (−479 ± 45 mV); voltage (1 V), frequency (1 Hz), duration (10 ms)	FAK/ERK signaling pathway; Ca^2+^ signaling pathway	Improved expressions of integrin β1, osteogenic differentiation	[82]
Chondrogenic	Cartilage	Bone marrow mesenchymal stem cells	Piezoelectric effects	Output voltage (451 mV), output current (17 μA)	Ca^2+^ signaling pathway, p38 MAPK signaling pathway	Activated voltage-gated calcium channels, chondrogenic differentiation, improved expressions of SOX-9, COL2A1, and ACAN	[54]
	Cartilage	Adipose-derived stem cells	Piezoelectric effects	Output voltage (33.7 mV)	TGF-β signaling pathway	Improved expressions of TGF-β1, chondrogenic differentiation, improved expressions of SOX-9, COL2A1, and ACAN	[83]
	Cartilage	Adipose-derived stem cells	Piezoelectric effects	Output voltage (3.6 V)	Ca^2+^ signaling pathway, TGF-β signaling pathway	Activated voltage-gated calcium channels, improved expressions of TGF-β1, chondrogenic differentiation, improved expressions of SOX-9, COL2A1, and ACAN	[84]
Neurogenic	Nerve system	Neural stem cells	Piezoelectric effects	Piezoelectric coefficient d_33_ (17.3 ± 9.3 pm/V)	Ca^2+^ signaling pathway	Neurogenic differentiation, improved expressions of NeuroD1, Lamb1, βIII-tubulin, and NeuN	[85]
	Nerve system	Bone marrow mesenchymal stem cells	Direct current electric fields	Electric field strength (8 ± 0.06 mV/mm)	Ca^2+^ signaling pathway	Neurogenic differentiation, improved expressions of nestin, βIII-tubulin, and MAP2	[86]
	Nerve system	Neural stem cells	Inductive electric fields	Rotating magnetic field (300 rpm), output voltage (0.59 mV), output current (21.8 μA)	Ca^2+^ signaling pathway, MAPK signaling pathway and cGMP-PKG signaling pathway	Activated voltage-gated ion channels, improved expressions of ChAT, GAD65, and c-Fos	[87]
	Nerve system such as spinal cord	Bone marrow mesenchymal stem cells	External electric fields	Electric field strength (100 mV/cm)	Ca^2+^ signaling pathway, PI3K/Akt/CREB signaling pathway, and MAPK signaling	Activated voltage-gated calcium channels, neurogenic differentiation, improved expressions of Tuj1 and PSD95	[88]
	Nerve system such as spinal cord	Neural stem cells	Inductive electric fields	Rotating magnetic field (500 rpm), output current (1.3 μA)	Ca^2+^ signaling pathway, PI3K/Akt signaling pathway, and MAPK signaling	Neurogenic differentiation, improved expressions of Tuj1 and MAP2	[90]
	Nerve system such as spinal cord	Neural stem cells	Inductive electric fields	Magnetic field (15 mT, 60 Hz, 50% duty cycle), output current (1.2 μA)	Calcium signaling pathway, PI3K-Akt signaling pathway, MAPK signaling pathway, and cAMP signaling pathway	Neurogenic differentiation, improved expressions of Tuj1 and BMP, enhanced axon growth	[89]
	Nerve system	Induced pluripotent stem cells	Alternating currents	Uniform electric field (±800 mV; 100 Hz)	Ciliary neurotrophic factor signaling pathway	Improved expression of ciliary neurotrophic factor, neurogenic differentiation, improved expression of Tuj1, MAP2, and Syn1	[91]
Cardiomyogenic	Heart	Induced pluripotent stem cells	Electric fields with monophasic square pulses	Electric field strength (5 V/cm), frequency (1 or 3 Hz), pulse duration (5 ms)	Ca^2+^ signaling pathway	Cardiomyogenic differentiation, improved expressions of ventricular genes (MYL2 and IRX4) and atrial genes (MYH6 and KCNA5)	[92]
	Heart	Induced pluripotent stem cells	Electric fields with biphasic square pulses	Electric field strength (1 V/cm), frequency (5 Hz), pulse duration (5 ms)	Ca^2+^/PKC/ERK signaling pathway	Activated voltage-gated calcium channels, cardiomyogenic differentiation, improved expressions of the cardiac transcription factors (HAND2 and TBX5) and construction genes (ACTCT1 and TNNT2 [cTnT])	[93]
	Heart	Mesenchymal stem cells	Pulsatile mechanoelectric cues	Frequency (1 Hz), voltage (3 V), short circuit current density (60 nA/cm^2^)	Autocrine growth factors (BMP-4, IGF, VEGF, and TGF-β) coupled intracellular signaling pathways and FAK/ERK signaling pathways	Improved expressions of BMP-4, IGF, VEGF, and TGF-β, cardiomyogenic differentiation	[94]
Other	Dental pulp	Dental pulp stem cells	Direct current electric fields	Electric field strength (150 mV/mm)	NOTCH signaling pathway, Ca^2+^ signaling pathway	Improved expressions of dentin phosphoprotein, Nestin, Reelin, and a-SMA	[95]
	Bone	Dental pulp stem cells	Piezoelectric effects	Output voltage (15 V), output current (12.6 μA), waveform (sinusoidal wave)	Notch signaling pathway, HIF-1 signaling pathway, MAPK signaling pathway, and cAMP signaling pathway	Construction of an immuno-angiogenic niche for bone regeneration by paracrine pattern	[96]
	–	Bone marrow mesenchymal stem cells	Nanosecond pulsed electric fields	Electric field strength (20 kV/cm or 10 kV/cm), frequency (1 Hz), duration (10 ns or 100 ns)	Epigenetic regulation	Enhanced multidirectional differentiation potential, instantaneously down-regulated DNA methylation transferase 1, demethylation of the promoters of stem cell pluripotency genes OCT4 and NANOG, improved expression of OCT4 and NANOG	[97]

#### Osteogenic differentiation

Electric stimulations can induce osteogenic differentiation of stem cells for bone regeneration. Electric stimulation activates voltage-gated calcium channels and promotes Ca^2+^ influx, and the improved Ca^2+^ concentration further activates the CaM/CaN/NFAT signaling pathway to promote the osteogenic differentiation of bone marrow MSCs (BMSCs) [77]. The induction to voltage-gated calcium channels may be correlated with the hemichannel externalization and the activation of Connexin 43, which allows the efflux of ATP to act to ATP receptors and depolarize cell membrane [45]. Improved intercellular Ca^2+^ concentration could also promote adenosine triphosphate (ATP) synthesis to induce actin remodeling by the conversion of G-actin to F-actin, ultimately improving the expression of bone morphogenetic protein 2 (BMP2) and promoting the osteogenic differentiation of BMSCs by the BMP2/Smad5 signaling pathway [78]. In addition, electric stimulation was also reported to promote Ca^2+^ influx and activate PI3K/Akt, WNT, and MAPK signaling pathways for the osteogenic differentiation of BMSCs [79]. In another study, improved intercellular Ca^2+^ concentration and activated PI3K signaling were correlated with the osteogenic differentiation of MSCs under electric stimulation [80]. Moreover, electric stimulation could improve the expression of PIEZO1 and PIEZO2, thus improving the response of BMSCs to mechanical stimuli [79]. Moreover, FAK/ERK signaling is another important signaling pathway for osteogenic differentiation of stem cells induced by electric stimulations, especially for surface potential or charge. In one study, the surface negative charge was also found to promote the expression of integrin α1, α2, α5, and β1, and then promoted the osteogenic differentiation of BMSCs by the FAK/ERK signaling pathway [81]. In another study, Shen et al. [82] also reported that the surface negative charge up-regulated the expression of integrin β1 and FAK but did not influence Ca^2+^ signaling, thereby promoting osteogenic differentiation of BMSCs by the FAK/ERK signaling pathway.

#### Chondrogenic differentiation

Electric stimulations can be used to induce the chondrogenic differentiation of stem cells for cartilage regeneration. Ca^2+^ influx by voltage-gated calcium channels involves the chondrogenic differentiation of BMSCs, and increased Ca^2+^ concentration further activates the p38 MAPK signaling pathway, ultimately improving the expression of cartilage formation markers including SOX-9, COL2A1, and ACAN [54]. In addition, electric stimulation has been shown to induce stem cells to secrete TGF-β1 to promote chondrogenic differentiation [83]. Another study reported that the secretion of TGF-β1 was correlated with Ca^2+^ influx by activated voltage-gated calcium channels under electric stimulation because the voltage-gated calcium channel inhibitor verapamil could apparently inhibit the chondrogenic differentiation of stem cells [84]. Therefore, voltage-gated calcium channel-mediated Ca^2+^ influx is also a critical factor for the chondrogenic differentiation of stem cells under electric stimulations.

#### Neurogenic differentiation

Electric stimulation can stimulate stem cells to undergo neurogenic differentiation for nerve system tissue regeneration. When electrical stimulation is applied to stem cells, Ca^2+^ influx is induced, thereby up-regulating Ca^2+^ signaling-sensitive NeuroD1 and promoting the expression of neural differentiation markers such as Nestin, βIII-tubulin, and NeuN [85,86]. The improved intercellular Ca^2+^ concentration is correlated with the activation of voltage-gated calcium channels, which then up-regulates p-CaMKII and induces MAPK and cGMP-PKG signaling pathways for the neurogenic differentiation of neural stem cells [87]. In studies on tissue engineering for spinal cord repair, Ca^2+^ influx by activated voltage-gated calcium channels could induce the PI3K/Akt and MAPK signaling pathways for the neurogenic differentiation of stem cells, showing great potential for the treatment of spinal cord injury [88–90]. In addition, electric stimulation has been reported to promote stem cells to produce ciliary neurotrophic factor to promote the neurogenic differentiation by autocrine [91].

#### Cardiomyogenic differentiation

Cardiomyocyte regeneration by the cardiomyogenic differentiation of stem cells is correlated with Ca^2+^ signaling [92]. Electric stimulation triggers the voltage-gated calcium channels to cause extracellular Ca^2+^ influx, and then the elevated intracellular Ca^2+^ level activates CaML and PKC/ERK signaling, ultimately promoting the differentiation of induced pluripotent stem cells into functionally mature cardiomyocytes [93]. In another study, pulsatile mechanoelectric cues that combine electric pulse and cyclic stretching were used to treat human MSCs, and researchers found that the simulations could promote the expression of some autocrine growth factors including BMP-4, IGF, VEGF, and TGF-β to promote the cardiomyogenic differentiation of MSCs by their intercellular signaling pathways [94]. In addition, pulsatile mechanoelectric cues could also induce FAK/ERK signaling pathways to improve the expression of GATA4 to promote cardiomyogenic differentiation [94].

#### Others

Under some special conditions, electrical stimulation does not lead to differentiation in a specific direction, but may lead to differentiation into multiple cells. As an example, when dental pulp stem cells (DPSCs) and HUVECs were cocultured, electric stimulation could induce DPMSs to differentiate into odontoblasts, pericytes, or smooth muscle cells [95]. Apart from differentiating into specific cell lineages, stem cells can be induced by electrical stimulation to promote tissue repair by orchestrating the tissue microenvironment. Under electric stimulation, multiple signaling pathways were activated in DPSCs including Notch signaling for immune regulation, HIF-1 signaling for angiogenesis, and MAPK signaling and cyclic adenosine monophosphate (cAMP) signaling for cytokine secretin, and all these signaling pathways allowed DPSCs to construct an immuno-angiogenic niche for bone regeneration [96]. Moreover, electric stimulation could enhance the multidirectional differentiation potential of MSCs by instantaneously down-regulating DNA methylation transferase 1 (DNMT1) to allow the demethylation of the promoters of stem cell pluripotency genes OCT4 and NANOG [97].

## Basic Fabrication to Simulate ECM Characteristics

**Fig. 3.**
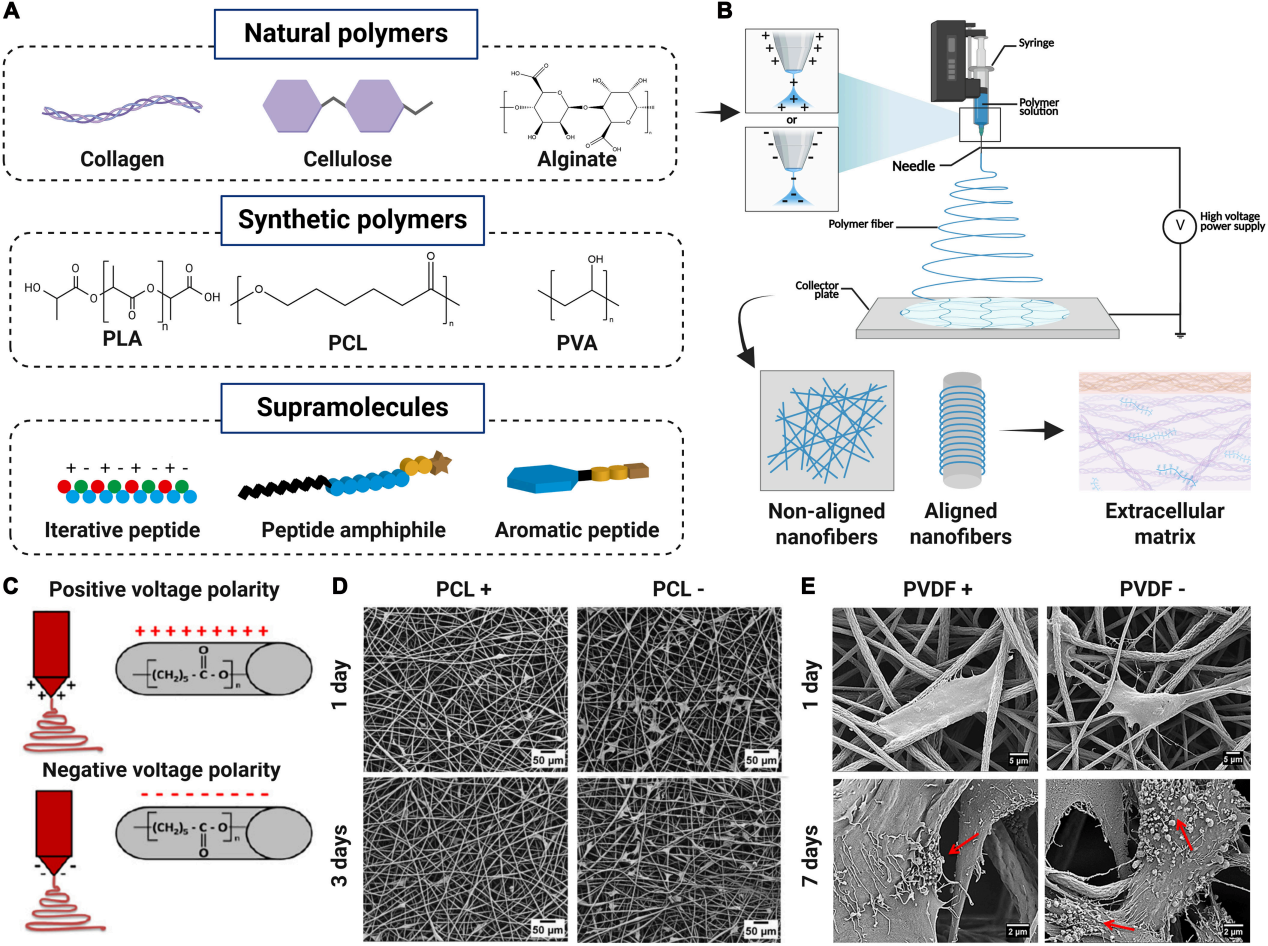
Basic fabrication of electrospun scaffolds to simulate ECM characteristics. (A) Distinctive electrospinning materials. (B) Electrospinning principle and fabrication of electrospun scaffolds with nonaligned or aligned nanofibers to simulate ECM. (C) Positive or negative voltage polarity to influence the surface potential of electrospun fibers by inducing polymer chain reorientation. Reproduced with permission [101]. Copyright 2020, Elsevier. (D) Enhanced cell proliferation and adhesion by PCL− electrospun scaffolds by negative voltage polarity. Reproduced with permission [102]. Copyright 2019, Wiley-VCH. (E) Improved collagen formation by PVDF− electrospun scaffolds by negative voltage polarity. Reproduced with permission [103]. Copyright 2019, ACS Publications.

Electrospinning can be used for tissue engineering to fabricate fiber-based scaffolds by a wide range of biomaterials including natural polymers, synthetic polymers, and supramolecules (Fig. 3A). Before electrospinning, the biomaterial is pre-prepared as a charged spinning fluid (usually a polymer solution or melt). Under a high-voltage electrostatic field, the spinning fluid forms a core jet from a spherical droplet when accumulated charges overcome the surface tension. The jet initially extends in a straight line and then is stretched and refined on account of bending instability in the electric field, with its diameter decreasing. Finally, the jet solidifies and deposits on the collector to form fiber-based scaffolds in the form of fiber film or mat (Fig. 3B). By selecting different collectors, electrospun scaffolds with nonaligned or aligned nanofibers could be fabricated to simulate ECM characteristics in different tissues (Fig. 3B). Moreover, the voltage polarity applied to the nozzle, positive or negative, could influence the properties of electrospun fibers from 3 aspects including the morphology, the surface, and the mechanical properties [98,99]. In particular, the voltage polarity could induce polymer chain reorientation and further modulate the surface potential on electrospun fibers, thus endowing them with certain electroactivity to regulate tissue regeneration [100]. When the positive voltage polarity is used, excess or net positive charges accumulated on the surface of the flying jet, which could attract electronegative chemical functional groups in the polymer chains to the jet surface. By contrast, the negative voltage polarity will repel these electronegative groups to the surface of the liquid jet due to accumulated negative charges. As a result, variations may occur in the surface potential of electrospun fibers (Fig. 3C) [101]. In general, cell adhesion and growth can be improved by the increased surface potential of electrospun fibers, which is beneficial for tissue regeneration [27]. For example, when polycaprolactone (PCL) was used for electrospinning, the positive voltage polarity attracted the negatively charged oxygen groups to the fiber surface, while the negative voltage polarity repulsed these groups. Consequently, PCL− fibers showed apparently improved surface potential compared with PCL+ fibers, and the results showed that increased surface potential on PCL− fibers could promote the formation of filopodia, cell proliferation, and the secretion of collagen for bone regeneration (Fig. 3D) [102]. In another study, the negative voltage polarity yielded polyvinylidene fluoride (PVDF) fibers with improved surface potential (−95 mV) by repelling the negatively charged fluorine groups, namely, PVDF− fibers, while the positive voltage polarity produced PVDF+ fibers with low surface potential (−173 mV) [103]. The results also showed that PVDF− fibers apparently promoted cell proliferation and promoted collagen formation and mineralization when compared with PVDF+ fibers (Fig. 3E) [103]. Therefore, the electroactivity by the voltage polarity is critical for optimizing tissue regeneration outcomes.

In addition to the modulation of collector configuration and voltage polarity to modulate the fiber alignment and surface potential, electrospinning materials and methods are the 2 other basic factors to simulate ECM structure and properties.

### Electrospinning materials

Considering that electrospinning is a technology for preparing fiber-based scaffolds through electric field forces, the basic electrospinning materials need to meet certain conditions. First, the basic materials either can be dissolved in an appropriate solvent to form a stable solution for fluid electrospinning or can melt at high temperatures and maintain thermal stability to avoid decomposition for melt electrospinning [104]. The conductivity of polymer solutions or melt is also important to fabricate scaffolds because the migration of charge in the electric field is the basis for the formation of jets. When conductivity is insufficient, ionic salts or conductive polymers can be added for adjustment to increase carrier concentration [105]. In addition, the polymer solution or melt should have a certain viscosity within an appropriate range. When it is too low, the surface tension is dominated by the flow of the jet easily breaks to form a bead-like structure; when it is too high, it is difficult to be stretched by the electric field to form fibers. Their surface tension needs to be controlled through solvent selection or additives to promote the stable formation of a Taylor cone and reduce the tendency to form a droplet [106]. Moreover, when applied in tissue engineering, the basic materials also need to be nonimmunogenic, nontoxic, highly biocompatible, and biodegradable, and the degradation products should be nontoxic and metabolizable. Thus, the screening of basic electrospinning materials requires comprehensive consideration of multidimensional factors such as processability, electrical properties, rheological characteristics, biocompatibility, and biodegradability, so that the materials can form a scaffold through electrospinning for tissue engineering [107].

Polymers are the mainstream electrospinning materials for tissue engineering on account of molecular chain intangibility, solvent processing flexibility, high performance tunability, biocompatibility, and biodegradability [108]. There are many ways to classify the polymers commonly used in tissue engineering, and they can be generally classified into 2 categories according to their origin, natural polymers/derivatives, and synthetic polymers [108]. On the one hand, natural polymers/derivatives such as collagen, gelatin, alginate, chitosan, hyaluronic acid, silk fibroin (SF), and decellularized ECM are biocompatible and biodegradable, and some of them are even biologically active to induce tissue regeneration due to cell-recognizable motifs [109]. When dissolved in an appropriate solvent, most of them could be electrospun to fabricate scaffolds for tissue engineering. However, the electrospinning solvents or electrospinning conditions may cause their denaturation [110], and only a few natural polymers/derivatives such as alginate could be electrospun when dissolved in a mild aqueous solution [111]. Moreover, scaffolds by natural polymers/derivatives are also limited due to poor mechanical properties, fast degradation rates, and potential immunogenicity. On the other hand, a wide variety of synthetic polymers could be used to fabricate scaffolds, including hydrophobic synthetic polymers such as polyvinyl alcohol (PVA) and polyethylene glycol as well as hydrophilic synthetic polymers such as PCL, polylactic acid (PLA), poly(lactic-co-glycolic acid) (PLGA), and poly(methyl methacrylate) [112]. The electrospun scaffolds obtained from synthetic polymers usually have excellent and tunable mechanical properties (including stiffness and elasticity), controlled degradation rates, and easy electrospinning, but there are some disadvantages including poor cell affinity (especially for hydrophobic polymers) and the lack of interfaces suitable for cell adhesion, diffusion, and proliferation.

In addition to polymers, supramolecular peptides with low molecular weight can be used as the basic materials for electrospinning. They could self-assemble or co-assemble to form nanofibers that are similar to polymer chains by noncovalent interactions, thus improving their electrospinnability. Various supramolecular peptide systems have been demonstrated to fabricate scaffolds by electrospinning, including ionic-complementary peptides [such as Ac-(LDLK)_3_-CONH_2_)] [113], peptide amphiphiles (such as C_16_-E_2_V_3_ and C_16_-V_3_A_3_E_3_) [114], aromatic peptide amphiphiles (such as Nap-FFKK, Pyr-FFKK, and TPP-FFKK) [115], and tyrosine-based ultrashort peptides (such as FY, YY, and WY) [116]. Similar to synthetic polymers, supramolecular peptides are also prepared by artificial synthesis, but they are preferable due to facial modification by functional motifs during peptide synthesis, thus improving the cell affinity to the electrospun scaffolds [8]. Therefore, supramolecular peptides have great prospects to fabricate scaffolds for tissue engineering by electrospinning. Moreover, electrospinning by supramolecular peptides can also be generalized to other small-molecule supramolecular systems with strong intermolecular interactions, providing broad prospects for the development of new electrospinning materials.

Conclusively, polymers and supramolecular systems are the main basic biomaterials for electrospinning to fabricate scaffolds, and they all show their unique properties. The rational selection of the biomaterials for electrospinning is critical to simulate target tissue for tissue regeneration. With the advancement of machine learning algorithms and the expansion of existing material databanks, artificial intelligence may help determine material composition and predict the scaffold properties by different surrogate models, apparently improving design efficiency and minimizing research costs [117].

### Electrospinning methods

Different electrospinning methods offer their distinct capabilities in fabricating engineering electrospun scaffolds with unique properties including fiber morphology, internal architecture, and scaffold structure, which would further influence biological outcomes such as cell adhesion, infiltration, proliferation, differentiation, nutrient/waste transport, and, ultimately, functional tissue regeneration. Therefore, this section details distinctive electrospinning methods (Table 2), highlighting how each technique uniquely contributes to tailoring basic electrospun scaffold for tissue engineering.

**Table 2.** Distinctive electrospinning methods to fabricate scaffolds

Category	Methods	Advantages	Disadvantages	Applications
Basic methods	Fluid electrospinning	Wide material applicability, easy morphology, easy functionalization by incorporating bioactive agents for tissue engineering	Residual solvents	Wound dressings, drug delivery, tissue engineering
Melt electrospinning	To avoid the usage of solvents	Only suitable for melt-processed polymers, limited functionalization, low fiber refinement, and thermal degradation	Tissue engineering
Modifications to fiber structure	Emulsion electrospinning	Formation of fibers with core–shell structure, simple and low cost, suitable for mass production	Uneven structure of bioactive agent, poor encapsulation effect	Core–shell fibers
Coaxial electrospinning	Precise control of fiber structure	High equipment costs and stringent requirements limit large-scale applications	Core–shell fibers, hollow fibers, porous fibers
Side-by-side electrospinning	Fabrication of scaffolds with a variety of properties for use (Janus fibers)	Complex process, difficult to accurately control fiber structure uniformity	Controlled release for multiple drugs
Sequential electrospinning	Capable of engineering hierarchically layered scaffolds, producing mats with controlled gradients, processing a wide spectrum of materials	Potentially poor interlayer interaction	Simulation of gradient tissue structures, co-delivery of different drugs
Modifications to fiber composition	Simultaneous electrospinning	Simple assembly of blended fiber networks, construction of architecturally graded systems, operation independent of material compatibility constraints	Demanding specialized collectors to prevent cross-interference	Endowing the scaffolds with multiple characteristics, mimicking in vivo-like gradients, multidrug delivery
Modifications to needles	Needleless electrospinning	High efficiency, avoiding needle blockage	Unstable jet	High-throughput manufacturing
Ultrasound enhanced electrospinning	Higher Young’s modulus (stiffness) and stress at break	Complexity of the selection of materials and equipment	Tissue engineering
Centrifuge electrospinning	High efficiency, controllable diameter	Fiber collection is difficult	High-throughput manufacturing
Modifications to collectors	Wet electrospinning	Increasing the porosity of the electrospun scaffolds	Complicated preparation process, poor mechanical properties	Construction of biomimetic porous scaffolds favoring cellular infiltration and nutrient transport
Precision deposition	Melt electrospinning writing	Precise deposition to create organized 3D structures	Limitations in the choice of thermoplastic materials, potential thermal degradation of bioactive components, high cost and complexity of equipment	High-resolution customizable mounts in tissue engineering
Near-field electrospinning	Adjustable fiber orientation control, broad material compatibility	Challenge to fabricate nanoscale fibers	Energy harvesting, tissue engineering

#### Basic electrospinning methods

**Fig. 4.**
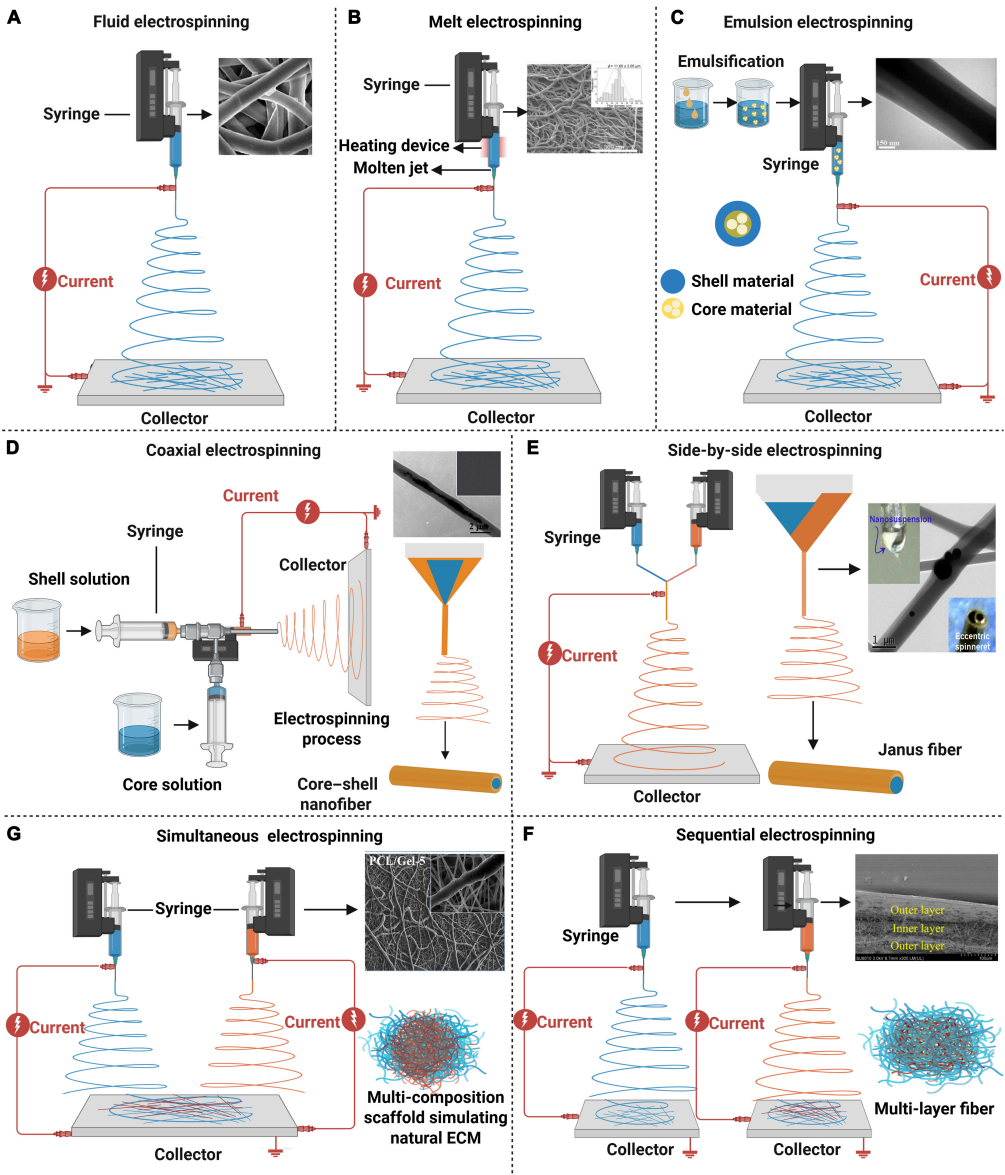
Basic electrospinning methods and modification to fiber structure and composition. (A) SEM images of P1-Hep-PCL/SF electrospinning fiber membranes prepared by fluid electrospinning [118]. Reproduced with permission. Copyright 2025, Elsevier. (B) SEM images of molten electrospinning polycaprolactone (PCL) fibers. Reproduced with permission [121]. Copyright 2019, MDPI. (C) TEM images of core–shell nanofibers prepared by emulsion electrospinning [122]. Reproduced with permission. Copyright 2024, Elsevier. (D) Typical TEM images of coaxial electrospinning fibers containing 5% CNT [127]. Reproduced with permission. Copyright 2016, Elsevier. (E) SEM images of HANUS fibers prepared by eccentric spinnerets. Reproduced with permission [131]. Copyright 2023, Elsevier. (F) SEM images of multilayer nanofiber membranes prepared by sequential electrospinning. Reproduced with permission [136]. Copyright 2020, Elsevier. (G) SEM images of PCL/Gel co-electrospinning films prepared by simultaneous electrospinning. Reproduced with permission [143]. Copyright 2021, Elsevier.

Electrospinning equipment typically consists of an injector connected to a syringe pump, a spinneret, a collector, and a high-voltage power supply. According to electrospun materials in different states (solution or melt), electrospinning can be categorized into fluid electrospinning and melt electrospinning. Fluid electrospinning is a widely used technique for fabricating nanofibers due to advantages including extensive material applicability, facile control over fiber morphology, and easy functionalization by incorporating bioactive agents for tissue engineering (Fig. 4A) [118]. The process is relatively straightforward and cost-effective, which allows us to produce continuous fibers from a wide range of polymer solutions and be easily scaled up for industrial applications [119]. However, concerns including solvent residue and environmental pollution should be taken into consideration. Thus, melt electrospinning has been developed to facilitate scaffolds without the use of solvents but is only suitable for melting polymers [120]. Although melt electrospinning could also be used to incorporate some bioactive agents, the bioactivity may be negatively influenced, and process parameters should be carefully controlled to guarantee their bioactivity (Fig. 4B) [121]. In addition, problems such as low fiber refinement and thermal degradation still need to be broken. Future research should determine the choice based on specific needs and material properties.

#### Modification to fiber structure and composition

Electrospinning generally promotes fibers with solid structures whose entire cross-section is composed of a single material. To modulate the structure of electrospun fibers, there are some electrospinning methods that have been developed, including emulsion electrospinning, coaxial electrospinning, and side-by-side electrospinning.

Emulsion electrospinning and coaxial electrospinning can be used to fabricate fibers with a core–shell structure, which allows the incorporation of bioactive agents in the core to promote tissue regeneration (Fig. 4C) [122]. During emulsion electrospinning, the continuous phase forms the outer shell of the fibers, while the dispersed-phase droplets are encapsulated in the interior, thus forming fibers with a core–shell structure [123]. The method has certain advantages, including simple operation, low cost, and suitability for mass production, but it is limited due to the high requirements for emulsion stability, which may result in an uneven structure and the unsatisfactory encapsulation of bioactive agents [124]. By contrast, coaxial electrospinning fabricates fibers with a core–shell structure by using an inner nozzle and an outer nozzle for the injection of 2 polymer solutions or melts, respectively [125]. Under the effects of an electric field, the inner material forms the core of the fiber, while the outer material is covered around the inner layer to form fibers with a core–shell structure [126]. Coaxial electrospinning allows precise control of the fiber structure, so it is suitable for the construction of multifunctional materials for tissue engineering, but the large-scale application is limited due to the high cost of the equipment and the strict requirements for solution (Fig. 4D) [127].

Side-by-side electrospinning can be used to fabricate Janus fibers by using a spinneret with 2 or more parallel needles to simultaneously eject different materials. Janus electrospun fiber is a type of nanofiber material with asymmetric properties on both sides. Compared with ordinary homogeneous materials, Janus materials can show good bifunctional synergy and can impart different characteristics to different parts of fibers [128]. Due to the advantages of the Janus structure, biologically active substances of different characteristics such as hydrophilic and hydrophobic polymers are often combined to achieve different functions such as drug delivery, multifunctional bone regeneration, and wound healing [129]. Side-by-side electrospinning can simultaneously spray different materials by using spinnerets with 2 or more parallel needles to prepare fibers with Janus structures, and accurately control the material composition, fiber morphology, and surface characteristics [130]. When preparing Janus fibers by side-by-side electrospinning, the design of the spinneret is very important because the port angle between the needles affects the width of the final Janus fiber [131]. Therefore, researchers have developed an eccentric nozzle for stably and continuously manufacturing Janus nanofibers; on the one hand, electrostatic energy is transferred more efficiently into the duplex compared to traditional side-by-side capacitors composed of 2 parallel metal capillaries. On the other hand, this eccentric nozzle provides a larger contact surface area for the duplex, ensuring an integrated treatment process [132]. For example, Yang et al. [131] used a parallel electrospinning process to prepare Janus wound dressing consisting of polyvinylpyrrolidone (PVP) and ethyl cellulose (EC) polymer matrix, in which ciprofloxacin and silver nanoparticles (AgNPs) were loaded on both sides (Fig. 4E). A continuous preparation process is maintained using homemade eccentric nozzles. Janus fibers have been shown to have good bactericidal activity in the growth of Gram-positive *Staphylococcus aureus* and Gram-negative *Escherichia coli*. In addition, based on the advantages of eccentric nozzles, the researchers have developed more Janus fibers based on side-by-side electrospinning [133]. These complex Janus fibers can provide an important platform for future complex tissues such as capillaries and nerve ending regeneration in muscles [134]. Although Janus fibers prepared by side-by-side electrospinning have unique advantages as tissue engineering scaffolds, their complex processes and the difficulty of precisely controlling the uniformity of fiber structures limit the widespread application of Janus fibers, and these problems need to be further solved in the future [135].

Electrospinning generally constructs scaffolds with the same type of fibers. To introduce different types of fibers into the same scaffolds, researchers have developed sequential electrospinning and simultaneous electrospinning. On the one hand, sequential electrospinning could be used to construct scaffolds with multilayered structure by one-by-one deposition of different materials, and the architecture of each layer could be easily controlled since there is only one jet fluid during scaffold fabrication (Fig. 4F) [136]. Multilayer scaffolds by sequential electrospinning can be used for the simulation of graded tissue structure [137] or interfacial tissue [138] and the distribution control of bioactive agents [139]. However, the inadequate interfacial adhesion among different layers is a major obstacle faced by sequential electrospinning, which may lead to a propensity for delamination. On the other hand, simultaneous electrospinning uses 2 or more spinnerets to concurrently extrude different materials for electrospinning on the same collector, thus integrating fibers from different materials to one scaffold [140]. The architecture of electrospun scaffolds by this method can be controlled by the collector selection and the spinneret placement. For example, scaffolds with a homogeneous architecture could be prepared by co-depositing different jet fluids to the middle position of a rotating mandrel [141], while scaffolds featuring a graded heterogeneous architecture could be fabricated by a facile misaligned deposition strategy, which involves adjusting the syringes at specific intervals along the mandrel’s length [142]. Considering that multiple materials are used, the method could well simulate the complex component and graded structure of natural ECM (Fig. 4G) [143]. Nevertheless, multiple jets from different spinnerets may impact each other; thus, irregular jet paths may make it difficult to obtain satisfied scaffolds.

#### Modification to needles

Conventional electrospinning faces inherent challenges in maintaining Taylor cone stability, as it is susceptible to perturbations from airflow fluctuations, needle dimensions, and viscosity-induced clogging. In addition, nozzle blockages frequently disrupt continuous fiber production during electrospinning. Therefore, the needles of the electrospinning device can be changed; thus, needleless electrospinning, centrifuge electrospinning, and ultrasonic enhanced electrospinning are invented.

**Fig. 5.**
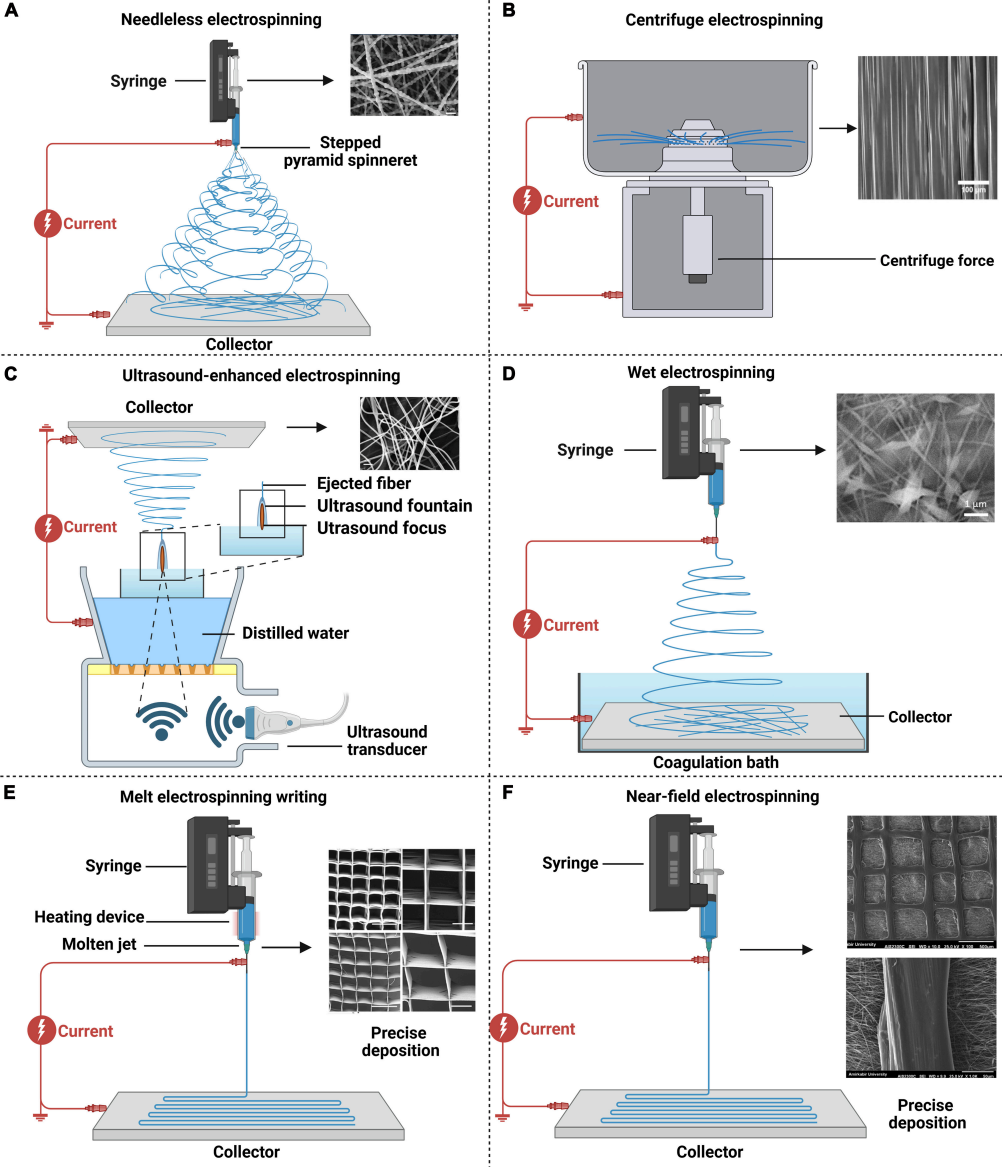
Modifications to needles, collectors, and precise deposition in electrospinning. (A) SEM images of PAN nanofiber with Si particle in the ratio by needleless electrospinning. Reproduced with permission [147]. Copyright 2022, Wiley-VCH. (B) SEM images of highly aligned fibers using centrifuge electrospinning. Reproduced with permission [149]. Copyright 2017, Elsevier. (C) SEM images of ultrasonic enhanced electrospinning nanofibers. Reproduced with permission [153]. Copyright 2019, MDPI. (D) SEM images of Alg/PEO/F127/GelF-MA 2D nanofibers were prepared using wet electrospinning. Reproduced with permission [154]. Copyright 2018, Elsevier. (E) SEM images of square and rectangular MEW microfiber scaffolds prepared by melt electrospinning writing. Reproduced with permission [158]. Copyright 2017, Wiley-VCH. (F) SEM image of microfiber composite scaffolds prepared with polycaprolactone (PCL)/hydroxyapatite (HA) using near-field electrospinning. Reproduced with permission [163]. Copyright 2025, Elsevier.

Compared with the traditional method, needleless electrospinning substantially improved productivity (from 1 to 18 ml·h^−1^) [144]. This technique acts as a fiber optic generator to load extremely high voltages and relies on the free surface of the liquid or designed bumps to simultaneously stimulate many jets. Needleless electrospinning systems employ 2 primary spinneret configurations: fixed and rotary [145]. In fixed spinnerets, externally applied forces—including magnetic fields, electric fields, or pneumatic flows—engineer surface protrusions that serve as nucleation sites for Taylor cone formation. Conversely, rotary spinnerets exploit high-voltage potentials to induce multiple electrohydrodynamic jets directly from their dynamic interfaces. Compared with traditional needle electrospinning, needleless electrospinning technology has higher production efficiency, which helps promote the application of electrospun nanofibers in multiple fields [146]. The core advantages of needleless electrospinning lie in large-scale production, high structural controllability, and biocompatibility, which are expected to promote the development of tissue engineering from single repair to functional tissue regeneration (Fig. 5A) [147].

Centrifugal electrospinning is another emerging electrospinning technique that utilizes rotational kinetics for the fabrication of electrospun fibers. In this technique, a cylindrical reservoir containing the polymer solution rotates at controlled angular velocities, subjecting the material to centrifugal forces. This actuates solution ejection from orifices while synchronized rotation of the nozzle and collector assemblies enables precise fiber diameter modulation through rotational speed optimization [148]. Compared with conventional electrospinning, centrifugal spinning prepares microfibers through centrifugal force, exhibiting distinctive advantages including no voltage, high production efficiency, and controllable fiber arrangement (Fig. 5B) [149]. The fiber materials it produces have a wide range of application potential in tissue engineering on account of their high specific surface area, porous structure, and exclusive mechanical properties such as elastic modulus, fracture strain, and adjustable hydrophilicity [150]. In recent years, the combination of centrifugal spinning and conventional electrospinning has become a research hotspot, such as preparing multilayer fiber or hybrid fiber mats through nuclear sheath structures to further enhance the functionality of the material [151].

Ultrasound-enhanced electrospinning has been developed to avoid the use of a nozzle, which could also promote the evaporation of solvent [152]. By contrast, high-intensity focused ultrasound bursts are employed to form a liquid protrusion from the surface of a polymer solution, and then a fiber stream will be induced from the protrusion to the collector when the polymer solution is charged with high voltage. The fiber diameter can be modulated by controlling the ultrasound parameters [153]. It has been shown that scaffolds by ultrasound-enhanced electrospinning showed higher Young’s modulus and higher rupture stress than those by conventional electrospinning (Fig. 5C) [153]. Although ultrasound-enhanced electrospinning has shown many advantages, it is limited by the choice of electrospinning materials and the utilization of complex equipment [153]. Therefore, further studies need to focus on technical optimization to improve applicability and efficiency and further explore its application potential in tissue engineering.

#### Modification to collectors

In addition to the innovation in needles, wet electrospinning has been developed to transform the collector from a conventional solid collector to a liquid bath collector. When electrospun fibers are deposited in the liquid reservoir, the wet medium helps fill the spaces between the fibers and prevent them from continuous packing, thereby improving the porosity of the electrospun scaffolds [154]. Therefore, wet electrospinning is suitable to construct biomimetic porous scaffolds that are conducive to cell infiltration and nutrient transport (Fig. 5D) [154]. However, the technique also has some shortcomings such as complex production processes and weak mechanical properties. Despite this, wet electrospinning still has broad application prospects for tissue regeneration that needs porous structure [155].

#### Precise deposition in electrospinning

To precisely control the deposition of fibers during scaffold fabrication, melt electrospinning writing (MEW) has been developed. It is an advanced electrospinning technology that utilizes molten thermoplastic polymers to fabricate fibers through high-voltage electrostatic fields, enabling precise control over fiber deposition via programmable collectors to create ordered 3D structures [156]. This solvent-free approach avoids issues of residual solvents and is particularly advantageous for tissue engineering, where it produces high-resolution, customizable scaffolds that mimic the ECM to promote cell alignment and tissue regeneration [157]. For example, Castilho et al. [158] used MEW to fabricate highly ordered microfiber scaffolds by a hydroxyl-functionalized polyester, and they found that cardiac progenitor cells are more efficiently arranged along the preferred direction of the fabricated scaffolds compared with traditional electrospinning-based scaffolds (Fig. 5E). In another study, the micropattern of curled fibers was developed by MEW to simulate the curled structure of collagen fibers in ligaments, and the scaffolds show great potential for tissue repair in bone–ligament interfaces [159]. While MEW offers exceptional structural control and biocompatibility, it is limited by material selection to thermoplastics, potential thermal degradation of bioactive components, and the high cost and complexity of equipment.

Near-field electrospinning (NFES) achieves precise control of the deposition position of fibers by shortening the distance between the nozzle and the receiving substrate and reducing the working voltage [160]. As a new tissue engineering scaffolding technique that combines 3D printing and electrospinning, NFES is used to create regular structures with smaller resolution and appropriate pores [161]. This technology shows great potential in the manufacture of functional scaffolds for tissue engineering, as it can simulate biological structures and support new tissue formation [162]. By precisely controlling the material systems required for cell behavior, 3D printing technology enables the creation of highly complex and multicomponent structures with clear architecture and composition. NFES technology is particularly suitable for the manufacture of biodegradable fibers with specific orientation arrangements, which is particularly important for guiding cell arrangements and tissue regeneration (Fig. 5F) [163]. Stents manufactured by NFES technology have excellent performance in mechanical properties, cytocompatibility, and cell support, demonstrating their promising potential in various applications including complex tissue regeneration scaffolds, porous tubes, and controlled drug delivery. In addition, NFES technology can also guide cell arrangement, proliferation, differentiation, generation of specific ECM, and tissue maturation by introducing biological activity cues [164]. The effects of these cues vary by material system, cellular phenotype, or potential synergistic interactions within multicue environments and therefore must be carefully selected according to the target application [165]. In summary, NFES technology has plenty of application prospects in fabricating scaffolds with specific biological activities and structural properties for tissue regeneration.

## Electroactive Fabrication to Simulate Bioelectric Microenvironment

**Fig. 6.**
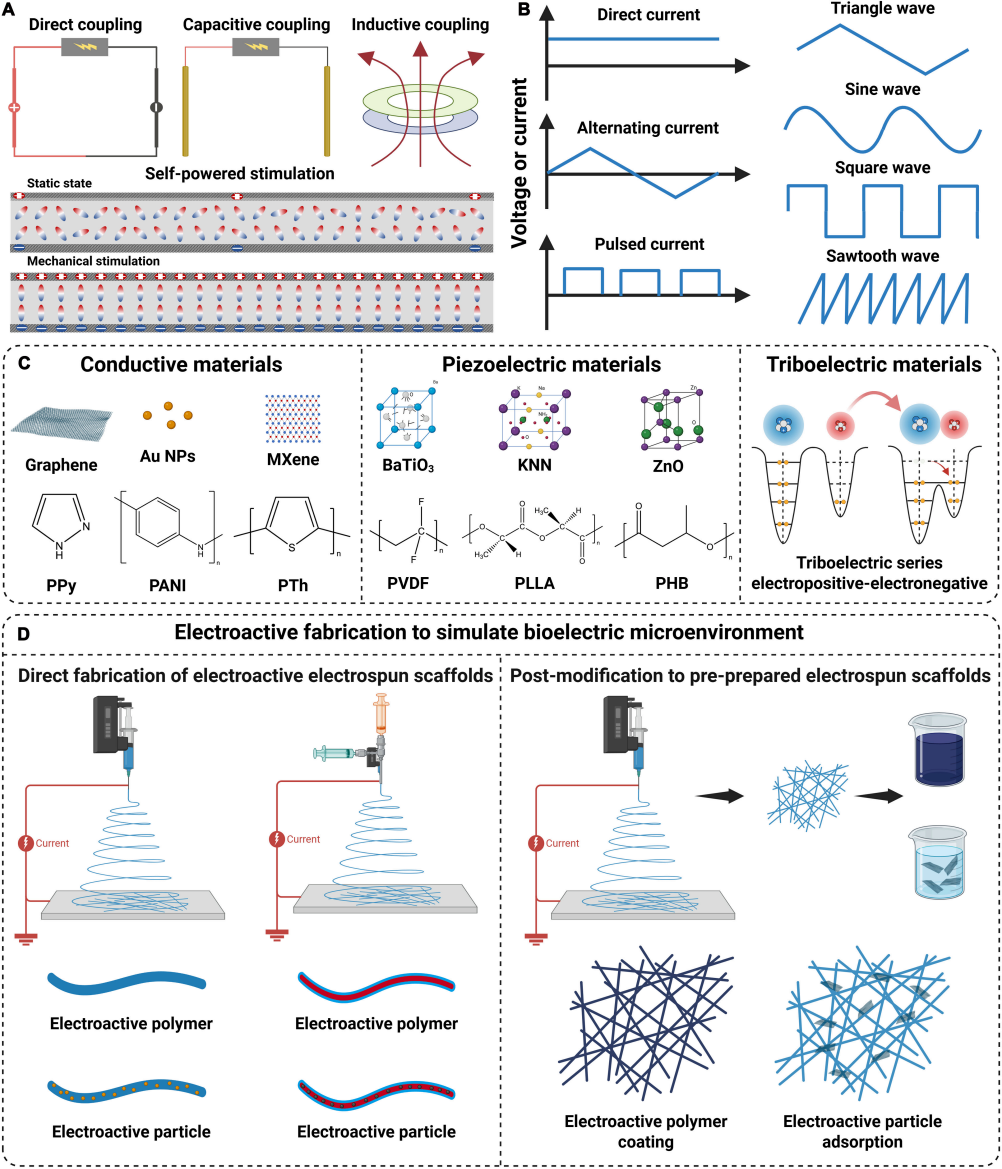
Electric simulations and electroactive fabrication by electrospinning to simulate bioelectric microenvironment. (A) Common electric stimulations including direct coupling, capacitive coupling, inductive coupling, and self-powered stimulation. (B) Common electrostimulation waveforms for tissue regeneration. (C) Conductive materials, piezoelectric materials, and triboelectric materials for electrospinning to fabricate conductive electrospun scaffolds. (D) Strategies of electroactive fabrication to simulate bioelectric microenvironment including direct fabrication and post-modification.

Various electric simulations have been developed to modulate cell behavior and promote tissue regeneration, and they can be divided into 4 groups including direct coupling, capacitive coupling, inductive coupling, and self-powered stimulation (Fig. 6A), and their features are summarized in Table 3. Direct coupling is a common experimental approach for applying electric stimulations in biological systems, and it involves placing conductive electrodes directly into the culture medium where they contact a conductive scaffold to deliver electrical signals [166]. Despite its simplicity and widespread adoption, this method faces substantial limitations related to undesirable effects including shifts in pH, localized heating, and the generation of toxic electrochemical by-products. By contrast, capacitive coupling and inductive coupling are 2 noninvasive stimulation techniques that can apply an electric field to cells or targeted tissues without direct physical contact [167]. For the capacitive coupling approach, 2 parallel disc-shaped electrodes are positioned on opposing sides and separated by an air gap without physical contact, which could improve biosafety due to its noninvasive nature [168]. However, a major limitation of capacitive coupling is its relatively low efficiency; thus, it often requires the application of higher voltages and extended stimulation durations to achieve the desired biological effects. In the inductive coupling technique, conductive coils or solenoids are positioned around the culture system or targeted tissues to generate a time-varying magnetic field, and this changing magnetic field induces an electromotive force according to Faraday’s law of induction, thereby creating an electric field perpendicular to the magnetic axis and prompting charge movement along potential gradients [169]. Although it mimics natural bioelectrical signaling and avoids direct electrode contact, its application remains limited due to the need for specialized devices and considerable resource investment. Moreover, unlike the first 3 driven by an external power source, self-powered stimulation does not require an external power source and is capable of generating electrical signals on its own by a distinctive mechanism such as piezoelectric effects or triboelectrification [170]. Although it possesses high biosafety and biocompatibility, it still faces major challenges such as unstable output, relatively low power, and imprecise control. In the form of current or voltage, there are monophasic or biphasic direct current (DC) and alternating current (AC) with distinctive waves including sinusoidal, square, triangle, and sawtooth waves, and there can be intervals in the waveform to form pulsed current (Fig. 6B).

**Table 3.** Comparison of common electric stimulation techniques

Features	Direct coupling	Capacitive coupling	Inductive coupling	Self-powered stimulation
Power source	External power	External power	External power	Ambient energy (self-sustaining)
Invasiveness	Invasive	Noninvasive	Noninvasive	Implantable or noninvasive
Biosafety	Low (harmful by-products)	High	High	Very high
Output stability	High (precise control)	High (precise control)	High (precise control)	Low (environment-dependent)
System complexity	Low	Medium	High (specialized equipment)	High (integrated system)
Primary application	In vitro studies, short-term stimulation	In vitro studies, transcutaneous stimulation	In vitro studies, deep tissue stimulation	In vitro studies, long-term implants, wearable devices

EAESs mainly exert their electroactivity through conductivity or self-generating power; thus, there are mainly 3 types of electroactive materials for electrospinning, including conductive materials, piezoelectric materials, and triboelectric materials (Fig. 6C). On the one hand, they can serve as raw materials for electrospinning to directly fabricate EAESs (Fig. 6D). On the other hand, they can be used to modify the surface of pre-prepared electrospun scaffolds, thereby imparting electroactivity (Fig. 6D).

### Electroconductivity

Some electric-sensitive tissues in the human body, such as nerves and heart, involve the conduction of electrical signals to perform their functions. Thus, distinctive conductive polymers and conductive nanomaterials can be used to endow electrospun scaffolds with electroconductivity to mediate charge transfer at the cell–substrate interface, promoting functional recovery and tissue regeneration (Table 4).

**Table 4.** Electroactive materials by electrospinning for tissue regeneration

Category	Material type	Advantages	Disadvantages	Target tissue/cell	Scaffold design	Ref.
Conductive polymers	Polypyrrole (PPy)	High electrical conductivity Facile synthesis Oxidation-reduction activity with superior biocompatibility	Prolonged degradation kinetics Chronic toxicological effects	Nerve tissue (PC-12 cells)	PELA-PPy fibers	[173]
Nerve tissue (PC-12 cells)				PLA-PPy fibers	[174]
Nerve tissue				PCL-PPy fibers	[175]
Nerve tissue				PPy-SF fibers	[176]
Nerve tissue				PPy-PLGA fibers	[177]
Polyaniline (PANI)	Structural polymorphism Economical fabrication Enhanced manufacturability Charge transport via doping mechanisms	Muscle tissue (C2C12 cells)		PANI-PLCL fibers	[180]
Muscle tissue (C2C112 cells)		PANI-PCL fibers		[178]
Poly(3,4-ethylenedixythiophene) (PEDOT)	Reduced interfacial impedance with optimal tissue compatibility	Nerve tissue (SCs and PC12 cells)		HEC-PEDOT fibers	[182]
Poly [2-methoxy-5-(2-ethylhexyloxy)-1,4-styryl] (MEH-PPV)	Mitigation of processability/solubility limitations in conventional conductive polymers Hierarchical porosity Biomolecular immobilization capability	Limited ionic conductance	Nerve tissue (PC-12 cells)	MEH-PPV-PCL fibers	[183]
Conductive nanomaterials	Multiwalled carbon nanotubes (MWCNTs)	Superior electrochemical performance and mechanical robustness	Suboptimal cytocompatibility with cytotoxic potential	Bone tissue (osteoblast)	PLA-MWCNT fibers	[189]
				Peripheral nerve tissue (SCs and BMSCs)	PCL-GELMA-MWCNTs fibers	[190]
	Carbon nanofibers (CNFs)	High specific surface area High aspect ratio High-quality conductivity Easy to function		Tendon tissue (tenocytes)	PLA/SF-CNF core–shell nanofiber	[194]
	Graphene family	Outstanding thermoconductivity Electrical conductivity Good biocompatibility		Nerve tissue	Graphene-SF fibers	[185]
				Peripheral nerve tissue (RSC96 cells)	rGO-GELMA/PCL fibers	[187]
				Nerve tissue (SCs and PC12 cells)	GO-PLA fibers	[186]
				Peripheral nerve regeneration (SCs and PC12 cells)	rGO-ApF/PLCL fibers	[188]
	Au nanoparticles (AuNPs)	High stability Good biocompatibility Easy to function	Inadequate colloidal stability Limited miscibility with polymeric matrices Oxidative degradation and surface passivation impair conductivity	Muscle tissue	Au-PLLA fibers	[195]
	MXene	High conductivity Hydrophilicity Exceptional mechanical properties Adjustable surface chemistry Good biocompatibility and biodegradability	Easy oxidation and insufficient mechanical strength	Bone tissue and angiogenesis	MXene-β-TCP	[201]
				Bone tissue and blood vessel network	PLLA-MXene	[202]
Piezoelectric ceramics	Barium titanate (BaTiO_3_)	High piezoelectric coefficient Good biocompatibility	Nondegradable under physiological conditions Thermolabile behavior	Bone and cartilage tissue	PCL/PANI-BaTiO_3_NPs	[209]
				Tendon tissue (macrophage1/2)	PCL-BaTIO_3_NPs	[210]
	Potassium sodium niobate (KNN)	High piezoelectric coefficient Environmentally friendly	Cellular toxicity Difficult processability	Spinal cord (NSCs and HUVECs)	PLA-KNN	[253]
	Zinc oxide (ZnO)	Biocompatible and biodegradable Antibacterial activity	Cellular toxicity Suboptimal piezoelectric response	Nerve tissue	PCL-ZnO	[211]
	Hydroxyapatite (HAp)	Biocompatible and biodegradable	Difficult to polarize Low poling efficiency Inferior mechanical integrity​	Bone tissue (BMSCs)	HAp/PVDF-TrFE	[208]
Piezoelectric polymers	Polyvinylidene fluoride (PVDF)/Polyvinylidene fluoride-trifluoroethylene (PVDF-TrFE)	High piezoelectric coefficient Biocompatible Easy to process	Nondegradable under physiological conditions	Bone tissue (rBMSCs)	PVDF fibers	[219]
				Wound healing (L929 fibroblast)	PVDF-TrFE fibers	[277]
	Poly-levolactic acid (PLLA)	Biocompatible and biodegradable Elastomeric behavior	Immunogenic inflammatory responses	Bone tissue (BMSCs, macrophage and HUVECs)	PLLA fibers	[80]
				Nerve tissue (NSCs)	PLLA fibers	[278]
				Nerve tissue (SCs)	PPy/PDA-PLLA	[220]
	Poly(3-hydroxybutyrate) (PHB)	Biocompatible and biodegradable Highly stable	Low piezoelectric coefficient Insoluble in water Poor mechanical properties	Bone tissue (osteoblasts)	CaCO_3_-PHB/PHBV	[221]
	Collagen	Biocompatible and biodegradable Low antigenicity	Poor mechanical strength Toxic crosslinking agents are often used	Bone, cartilage, and skin tissue engineering Drug delivery	HAp-collagen	[224]
	Cellulose	Biocompatible and biodegradable Excellent cell adhesion	Minimal piezoelectric performance Restricted pore dimensions	Bone and neural tissue engineering Drug delivery	–	[216]
	Chitosan	Noncytotoxic High porosity	Low piezoelectric coefficient `Poor mechanical strength	Bone, cartilage, and skin tissue engineering Drug delivery Anticancer agent	–	[223]
Triboelectric polymers	PVDF	Electronegative polymer Containing many fluorine groups with a strong contact electrification effect	Nondegradable under physiological conditions	Wound healing	PVDF/polyurethane	[236]
Polyamide	Electroactive polymer High electron-donation affinity	Nondegradable under physiological conditions	Wound healing	Co-polyamide fibers	[237]
PCL	Electronegative polymer Biocompatibility FDA approved Good mechanical properties	Slow degradability Hydrophobicity Acidic degradation products	Wound healing	PCL fibers	[238]
PLGA	Electroactive polymer Biocompatibility Degradability FDA approved	Acidic degradation products Weak mechanical properties	Wound healing	PLGA fibers	[238]
PHB	Electropositive polymer Biodegradability	Insoluble in water Poor mechanical properties	Vascular grafts	PHB fibers	[239]

#### Conductive polymers

**Fig. 7.**
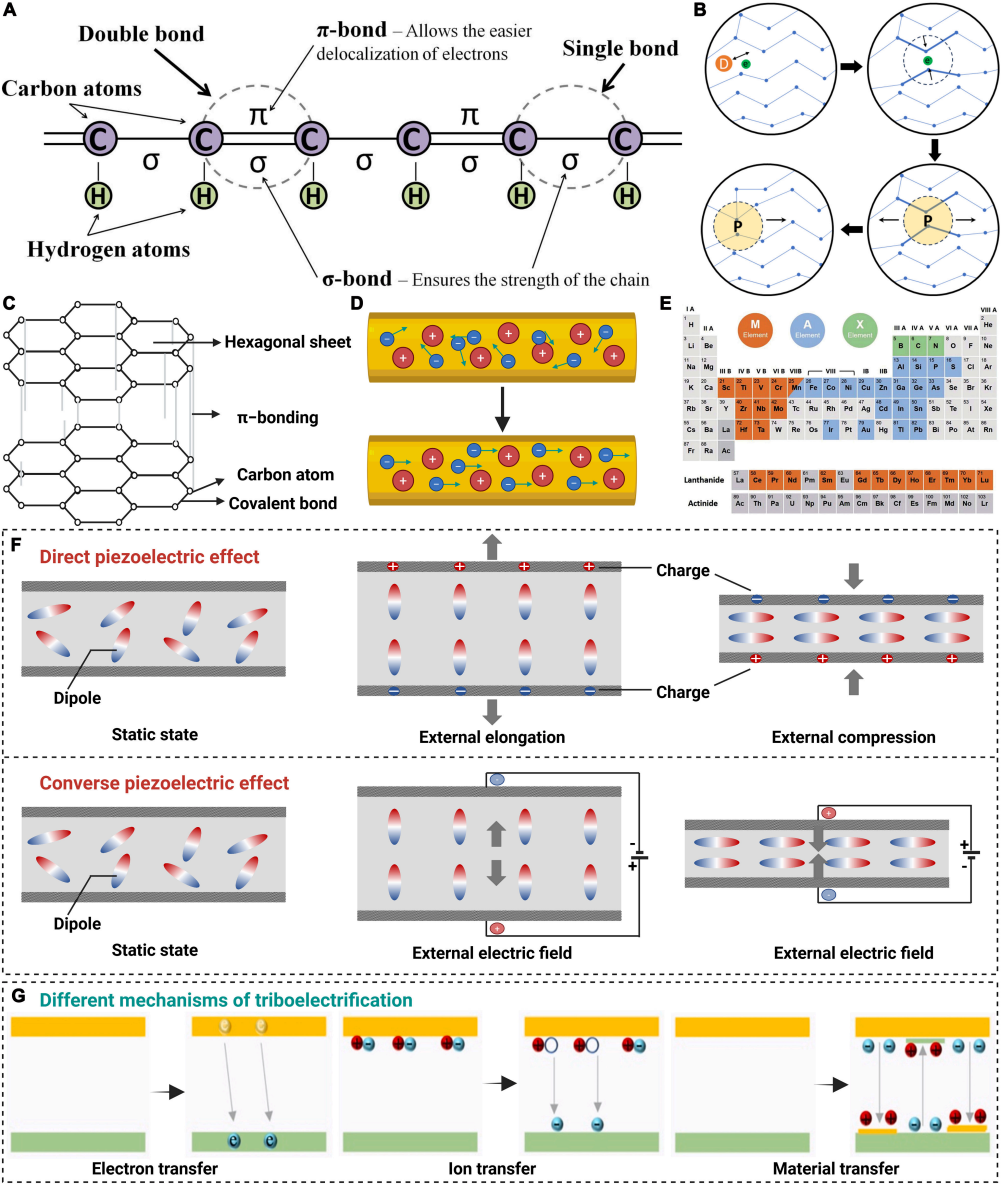
Mechanisms for electroconductivity, piezoelectric effects, and triboelectrification. (A) Schematic illustration of the backbone of conductive polymers. Reproduced with permission [172]. Copyright 2014, Elsevier. (B) Mechanisms for the electroconductivity of conductive polymers. Reproduced with permission [172]. Copyright 2014, Elsevier. (C) Mechanisms for the electroconductivity of carbon-based graphite. (D) Mechanisms for the electroconductivity of metal nanomaterials. (E) Element distribution of MXene with its chemical formula M_*n*+1_X*_n_*T*_x_*. Reproduced with permission [199]. Copyright 2021, Wiley-VCH. (F) Piezoelectricity in the nature including direct piezoelectric effect and converse piezoelectric effect. (G) Different mechanisms of triboelectrification including electron transfer, ion transfer, and material transfer. Reproduced with permission [230]. Copyright 2021, Elsevier.

As novel organic materials, conductive polymers demonstrate electrical and optical performance similar to metals and inorganic semiconductors, while also featuring superior flexibility and favorable processing properties [171]. The backbone of conductive polymers is composed of alternating single bonds (σ bonds) and double bonds (π bonds), with p-orbitals of carbon atoms to overlap each other to form a delocalized π molecular orbital spanning multiple atoms (Fig. 7A) [172]. Doping is the critical step in imparting conductivity, essentially changing the electronic structure through redox reactions. When an electric potential is applied, the doped polarons/double polarons move within and between chains to achieve conductivity (Fig. 7B) [172]. For tissue regeneration, there are various conductive polymers that can be used for electrospinning, including polypyrrole (PPy), polyaniline (PANI), polythiophene (PTh), poly(3,4-ethylenedixythiophene) (PEDOT), and poly [2-methoxy-5-(2-ethylhexyloxy)-1,4-styryl] (MEH-PPV).

PPy shows high conductivity on account of p-type bipolaron conduction, coupled with interchain electron hopping and ionic motion of anions or cations. It has been widely used to fabricate conductive scaffolds for tissue regeneration due to high biocompatibility and electro-responsivity. For example, Zhou et al. [173] prepared nerve catheters made from poly(d,l-lactide)-copoly(ethylene glycol) and PPy by electrospinning and used them to regenerate peripheral nerve defects. In another study, Tian et al. [174] used electrospun electrically conductive oriented nanofibers based on PLA and PPy for neural tissue engineering and showed that the oriented nanofibers were better able to conduct electricity than random fibers. In addition, PPy can be coated to the surface of electrospun scaffolds to improve electroconductivity [175]. For example, PPy-coated SF fibers have been shown to support adhesion and proliferation of MSCs and fibroblasts [176]. In another study, PPy was coated on PLGA fibers and has been shown to allow the growth and differentiation of PC-12 cells and hippocampal neurons [177]. However, PPy could increase the hydrophilicity of EAESs, which negatively impacts the tissue regeneration.

PANI is another common conductive polymer for tissue engineering, which shows advantages including low cost, high stability, and adjustable oxidation state. Generally, PANI is blended with other electrospinning materials to fabricate EAESs [178]. For example, PANI has been electrospun with PCL to produce substrates for heart and skeletal muscle tissue engineering [179,180]. Nanofibers of PANI-poly(L-lactide-co-epsilon-caprolactone) (PLCL) were also electrospun and reportedly supported adhesion of human dermal fibroblasts, NIH-3T3 fibroblasts, and C2C12 myoblasts [181]. However, PANI may cause chronic inflammation due to the potential toxicity that impacts its further clinical translation.

PTh and its derivative PEDOT could be also used to fabricate conductive electrospun scaffolds, and PEDOT shows broader application prospects than PTh because it exhibits enhanced conductivity and improved electrochemical stability on account of a dioxyalkylene bridge spanning the 3- and 4-positions of its heterocyclic ring. As an example, Song et al. [182] designed a helical structure electrospinning conductive conduit for peripheral nerve regeneration through simultaneous electrospinning, and the conductivity of the scaffold was substantially enhanced by PEDOT. The results showed that this conductive conduit could apparently enhance the adhesion and proliferation of SCs and PC12 cells, and further promote the expression of neurorelated proteins under electric stimulations [182].

While conductive polymers showed great potential to fabricate conductive electrospun scaffolds, the poor solubility derived from the rigid backbone blocks their applications. In contrast, MEH-PPV shows superior solubility in common electrospinning solvents, indicating good processability to fabricate conductive electrospinning scaffolds [183]. In addition, unsatisfactory biodegradation is still a critical challenge to hinder its clinical translation; thus, the next generation of biodegradable conductive polymers should be developed for tissue engineering.

#### Conductive nanomaterials

There are mainly 3 categories of conductive nanomaterials to be incorporated into electrospun scaffolds to achieve electroconductivity, including carbon-based nanomaterials, metal nanomaterials, and MXene.

Carbon-based nanomaterials are mainly derived from graphite, a layered mineral in nature with certain conductivity. Each layer of carbon atoms of graphite forms a planar hexagonal honeycomb structure through an sp^2^ hybrid orbital, and each carbon atom is connected with 3 adjacent carbon atoms at covalent bonds. At the same time, the remaining unbonded p-orbital electrons of each carbon atom are perpendicular to the plane, forming a delocalized large π bond (π electron cloud) in the layer (Fig. 7C). These electrons are not bound by a single atom and can move freely, giving them certain conductivity. Based on graphite, researchers have developed graphene with a single-layer structure to share similar conductive mechanisms, but the conductivity of graphene is substantially enhanced because its one-dimensional structure can avoid interlayer electron scattering [184]. In one study, graphene has been blended with SF to fabricate aligned conductive scaffolds for nerve tissue engineering [185]. In addition, graphene oxide (GO) and reduced graphene oxide (rGO) are 2 important derivatives of graphene with their surface containing functional groups; thus, the 2 derivatives are mainly used to construct conductive electrospun scaffolds for tissue regeneration [186,187]. For example, Wang et al. [188] coated rGO onto ApF/PLCL nanofiber scaffolds, and the results revealed that PC12 cells cultured on conductive electrospun scaffolds showed high differentiation. Given the high conductivity of the graphene family, they are expected to show their potential in endowing electrospun scaffolds with electroconductivity, but it is highly recommended to take the potential toxicity into account to improve biocompatibility.

Carbon nanotubes (CNTs) are seamless cylinders curled by hexagonal lattice made of sp^2^ hybrid carbon atoms, and they maintain the characteristics of large π bond delocal electrons in axial direction to provide free-moving carriers, exerting conductive properties. There are mainly 2 types of CNTs including single-walled CNTs and multiwalled CNTs (MWCNTs), among which MWCNTs have been widely used due to high conductivity, enhanced mechanical properties, and biocompatibility after modification. For example, Shao et al. [189] successfully manufactured randomly oriented and aligned conductive nanofibers of biodegradable PLA through an electrospinning process, where MWCNTs are embedded. These conductive nanofiber webs provide a unique system to study the synergistic effects of topographic cues and electric stimulations on osteoblast growth. In neural tissue regeneration engineering, Hu et al. [190] combined amine-functionalized MWCNTs with PCL and gelatin to create aligned or randomly conductive nanofibers by electrospinning. The study found that conferring conductivity to aligned nanofibers substantially enhances their ability to promote differentiation of SCs and could induce alignment of BMSCs to promote peripheral axon regeneration.

Carbon nanofibers (CNFs) are one-dimensional nanostructured carbon materials characterized by their high specific surface area, high aspect ratio, and high-quality conductivity [191]. CNFs consist of curved and stacked graphene sheets arranged in various orientations, showing sp^2^-based hybridization in a diameter from 50 to 200 nm [192]. Their exceptional electrical conductivity facilitates efficient propagation of bioelectrical signals, rendering them particularly suitable for tissue engineering [193]. For example, Yu et al. [194] wrapped conductive, high-strength, loose single bundles of CNFs in a nanofiber membrane in a tendon repair study to create an electrospun scaffold containing CNFs. The carbon fiber monofilament used in the study had a diameter of 5.07 ± 1.20 μm, which matched the diameter of tendon collagen, which could quickly establish the connection between tendon tissue and the stent, and better promote the recovery of electrical signal pathways.

Metal nanomaterials also show excellent electron conductivity because they contain a large number of freely moving electrons, which move irregularly when there is no electric field; when an external electric field is applied, the electrons are driven by the electric field force to form directional movement, thereby generating an electric current (Fig. 7D). Among various metal nanomaterials, gold nanoparticles (AuNPs) and AgNPs have been widely used in tissue engineering [195,196]. For example, researchers blended poly(L-lactic acid) (PLLA) with AuNPs and prepared a biodegradable nanofiber scaffold through electrospinning technology, and the experimental results show that the nanofiber scaffold has good conductivity and degradability and biocompatibility [195]. Compared with AgNPs, AuNPs showed high stability and biocompatibility, controllable morphology and dispersed sizes, and easy surface functionalization [197]. In contrast, AgNPs are mainly used to exert antibacterial activities, especially for infected wound healing [198].

MXene, namely, transition metal carbides/nitrides, is a new type of 2-dimensional (2D) inorganic compound nanomaterial, and its chemical formula is M_*n*+1_X*_n_*T*_x_* (where M represents early transition metals such as Ti, Nb, etc.; X represents carbon/nitrogen; and T*_x_* denotes surface functional groups such as -OH, -F, =O, etc.), as illustrated in Fig. 7E [199]. MXene exhibits unique properties including high conductivity, hydrophilicity, exceptional mechanical properties, and adjustable surface chemistry, showing great potential to be used to endow electrospun scaffolds with electroconductivity in tissue engineering [200]. For example, Zhang et al. [201] blended MXene and β-TCP into poly(lactic acid-trimethylene carbonate) for electrospinning, thus fabricating conductive electrospun scaffolds for bone regeneration. The results showed that MXene apparently improved scaffold electroconductivity and could mediate the external electric stimulation to promote angiogenesis and bone regeneration [201]. In another study, the addition of MXene into PLLA electrospun solutions apparently enhanced the surface potential of electrospun scaffolds (−415.7 mV) when compared with rGO-incorporated PLLA scaffolds (−60.4 mV) and PLLA scaffolds (−9.3 mV) [202]. Additionally, the introduction of MXene induced smaller, more uniform agglomerates, while the incorporation of rGO promoted larger and more variable clusters [202]. Both surface potential and agglomeration patterns modulated interfiber spacing, and MXene showed superior cell migration and scaffold properties over rGO [202]. Therefore, MXene is a new generation of conductive nanomaterials for tissue engineering when compared with other conductive nanoparticles, which showed improved biocompatibility, biodegradability, and electroactivity.

### Piezoelectric effects

Piezoelectricity is very common in nature, with its core lying in the noncentrosymmetric crystal structure within piezoelectric materials [203]. This structure causes the centers of positive and negative charges in the piezoelectric material not to coincide in the absence of external force or electric field, resulting in an inherent electric dipole moment. When piezoelectric materials are subjected to external mechanical stress and undergo deformation, internal electrical polarization occurs (a change in electric dipole moment), leading to the generation of opposite charges on opposing surfaces of the material. This phenomenon is known as the direct piezoelectric effect or piezoelectric effect (Fig. 7F). In contrast, when an external electric field is applied to a piezoelectric material, internal electrical polarization occurs, causing mechanical deformation in the material, which is referred to as the converse piezoelectric effect (Fig. 7F). In the field of tissue engineering, the direct piezoelectric effect is often utilized, and the constructed piezoelectric scaffolds can be regarded as an AC voltage source that generates local transient currents to promote tissue regeneration [204]. Distinctive piezoelectric ceramics and piezoelectric polymers can be used for electrospinning to fabricate piezoelectric electrospun scaffolds for tissue regeneration (Table 4).

#### Piezoelectric ceramics

There are mainly 2 types of piezoelectric ceramics including lead-based and lead-free piezoelectric ceramics. Lead-based piezoelectric ceramics such as lead zirconate titanate (PZT) are typically not used in tissue regeneration because of their toxicity. In contrast, lead-free piezoelectric ceramics such as barium titanate (BaTiO_3_) [205], zinc oxide (ZnO) [206], potassium sodium niobate (KNN) [207], and HAp [208] have been used for tissue regeneration on account of their excellent piezoelectric properties and certain mechanical properties. In general, these ceramics are combined with other basic polymers to fabricate piezoelectric electrospun scaffolds for tissue engineering [209]. For example, Dai et al. [210] used coaxial electrospinning to fabricate a self-powered piezoelectric electrospun scaffold by PCL (outer shell) and BaTiO₃ (inner core), and the results showed that the piezoelectric signals generated by the scaffold could promote tendon regeneration by multiple pathways including inflammation inhibition, tenogenic differentiation, and the reduction of lipid deposition. In another study, Mao et al. [211] developed piezoelectric electrospun scaffolds for peripheral nerve regeneration by co-electrospinning ZnO completely dissolved in PCL solution, and the results showed that ZnO-loaded electrospun scaffolds showed faster and more effective sciatic nerve repair in vivo relying on the piezoelectricity of ZnO. Despite the potential to fabricate piezoelectric electrospun scaffolds, piezoelectric ceramics in tissue engineering are limited by the potential toxicity and the high synthesis temperatures (usually >600 °C) that are usually required to perform polarization [212]. These problems still need to be addressed in future research.

#### Piezoelectric polymers

Compared with piezoelectric ceramics, piezoelectric polymers are generally not cytotoxic and have improved mechanical properties and enhanced deformation capabilities, which makes them useful not only in simulating the characteristics in hard tissues, but also in soft tissues [213]. PVDF and its derivatives PVDF-TrFE, PLLA, poly(3-hydroxybutyrate) (PHB), collagen, cellulose, and chitosan are the most used piezoelectric polymers and have many applications in bone, nerve, and cardiac tissue regeneration.

PVDF is a semicrystalline polymer exhibiting 50% to 60% crystallinity, characterized by a molecular structure of repeating -(CH₂-CF₂)- units with polar fluorine moieties. Its piezoelectric properties arise from 5 distinct crystalline polymorphs (α, β, γ, δ, and ε) featuring divergent dipole alignments, among which the α- and β-phases demonstrate predominant technological relevance [214,215]. The β-phase PVDF is particularly distinguished by its exceptional piezoelectric voltage coefficient (*g*_33_), coupled with intrinsic flexibility, facile processability, robust chemical resistance, mechanical durability, and biocompatibility. These attributes collectively establish β-PVDF as the preeminent piezoelectric polymer for electrospun scaffold fabrication [216]. Furthermore, this thermoplastic enhances cellular adhesion and proliferation through tunable surface characteristics—modulated by polarization state, surface energy, and scaffold architecture. Notably, polarized PVDF surfaces demonstrate substantial osteogenic potential, where piezoelectric stimulation promotes MSC differentiation and accelerates mineralized matrix deposition through electromechanical signaling pathways [217]. Moreover, PVDF-TrFE, a copolymer obtained from vinylidene fluoride (VDF) and trifluoroethylene (TrFE) monomers, exhibits superior piezoelectric coefficients relative to all known organic piezoelectric polymers. It maintains the biocompatibility of PVDF homopolymers while demonstrating enhanced cell adhesion and proliferation [218]. Consequently, PVDF-TrFE serves as a critical biomimetic platform for recapitulating the electromechanical microenvironment of native piezoelectric tissues [219].

PLLA is another flexible piezoelectric polymer to fabricate scaffold by electrospinning, exhibiting superior biodegradability and biocompatibility when compared with PVDF [80,220]. PHB is also a commonly used piezoelectric polymer with a relatively long degradation time; thus, PHB-based scaffolds could provide support for long-term tissue regeneration [221]. Overall, although PLLA and PHB can be used to develop piezoelectric electrospun scaffolds, their low voltage coefficients might limit their applications when compared with PVDF and PVDF-TrFE.

In addition to synthetic piezoelectric polymers, some nature polymers, such as collagen, cellulose, and chitosan, show piezoelectric properties. Collagen delivers biologically instructive cues that facilitate diverse cellular responses, and it could mediate piezoelectric effects due to the central symmetric structure and the presence of multiple molecular dipoles [222]. To enhance its piezoelectric performance, hydrolyzed collagen that is achieved through deamidation of asparagine/glutamine residues has emerged as a promising alternative for tissue regeneration [223]. It could be functionalized with some piezoelectric ceramics such as HAp to augment charge generation capacity [224]. Cellulose is a glucose homopolymer interconnected via β(1→4) glycosidic bonds exhibiting exceptional biocompatibility and high mechanical tensile, and it also shows a small shear piezoelectric charge coefficient of 0.1 pC/N [225]. In addition, chitosan, a deacetylated chitin derivative, shows a limited piezoelectricity (*d*_25_ = 0.2 to 1.5 pC/N), but it shows advantages including biocompatibility and antibacterial capacities [223]. Although the abovementioned natural polymers are not widely used in fabricating piezoelectric electrospun scaffolds, they could also be used as basic electrospun materials, thus endowing the scaffold with certain piezoelectricity.

Notwithstanding superior processing flexibility, piezoelectric polymers exhibit substantially inferior piezoelectric coefficients relative to piezoelectric ceramics. In addition, synthetic piezoelectric polymers are limited by their abiotic degradability and cytotoxicity when used for a long time in tissue regeneration. Therefore, combining piezoelectric polymers with piezoelectric ceramics with superior piezoelectricity or other biocompatible materials is a promising strategy to overcome the drawbacks of piezoelectric polymers [226,227]. In addition, enhanced electroactivity for tissue regeneration can be realized by combining the piezoelectric effect with the surface potential of electrospun scaffolds, and the surface potential determined by the voltage polarity can also enhance the piezoelectricity of piezoelectric polymer-based electrospun scaffolds [228,229].

### Triboelectrification

Triboelectrification is another common phenomenon for generating electrical signals. Unlike piezoelectric effects, the core principle of triboelectrification is the transfer of charge between 2 dissimilar materials with differing electronegativities. Due to the varying abilities of different substances’ atomic nuclei to bind outer electrons, the material with weak nucleus binding ability loses some electrons during contact and friction, and these electrons are transferred to the material with strong nucleus binding ability, resulting in the 2 materials acquiring equal amounts of opposite charges (Fig. 7G) [230]. In addition to the electron transfer theory to explain the mechanism of triboelectrification, there are also other mechanisms such as ion transfer and electron transfer to elucidate the complex process (Fig. 7G) [230]. Ion transfer is a mechanism in triboelectrification where mobile ions, rather than electrons, move between surfaces during contact, and this process is particularly relevant for insulators where free electrons are absent [231]. Additionally, material transfer involves the exchange of tiny material fragments between surfaces upon friction, and the transfer indicates that covalent bonds in polymer chains break, generating reactive free radicals that could form charged species by the reaction with oxygen or water [232].

Almost all materials possess triboelectric properties, so triboelectric series have been proposed to provide an empirical predictive framework for the nature of the charge a material acquires after friction [233]. When any 2 materials in the series rub against each other, the material positioned at the top of the series tends to lose electrons and become positively charged, while the material near the bottom of the series tends to gain electrons and become negatively charged [234]. Additionally, the greater the distance between 2 materials in the series, the greater the amount of charge usually generated upon friction, and the more pronounced the electrostatic effect [234]. A series of polymers have been applied for electrospinning to fabricate electrospun scaffolds with triboelectrification properties, and they can be divided into electronegative polymers (such as PVDF, PVDF derivatives, PVC, PI, PAN, PLA, and PCL) and electropositive polymers (such as polyamide, PHB, PVP, PVA, EC, and SF) according to their triboelectric polarity [235]. When used for tissue engineering, both electroactivity and biocompatibility should be taken into consideration, and some triboelectric materials have been used for electrospinning to promote tissue regeneration (Table 4). For electronegative polymers, PVDF has been used to fabricate electrospun membranes because it contains many fluorine groups with a strong contact electrification effect [236]. For electropositive polymers, polyamide is generally used because it shows high electron-donation affinity [237]. In recent years, studies have focused on the application of degradable polymers to fabricate triboelectric electrospun membranes for tissue engineering. In one study, PCL, a Food and Drug Administration (FDA)-approved biodegradable medical material, was used as an electron acceptor for electrospinning because its hydrophobic alkyl chain structure readily captures electrons [238]. Correspondingly, PLGA, with a certain degree of degradability, was used as an electron donor for electrospinning because its backbone contains hydrophilic ester groups that easily lose electrons [238]. In another study, PHB, a biodegradable biopolymer with excellent biocompatibility, was used as an electropositive material for electrospinning because it possesses oxygen-rich functional groups with weak electron affinity [239]. Therefore, biocompatible and degradable triboelectric polymers show great promise for tissue engineering, which help avoid potential side effects and the risks associated with secondary surgical removal.

## Smart Applications in Tissue Regeneration

**Fig. 8.**
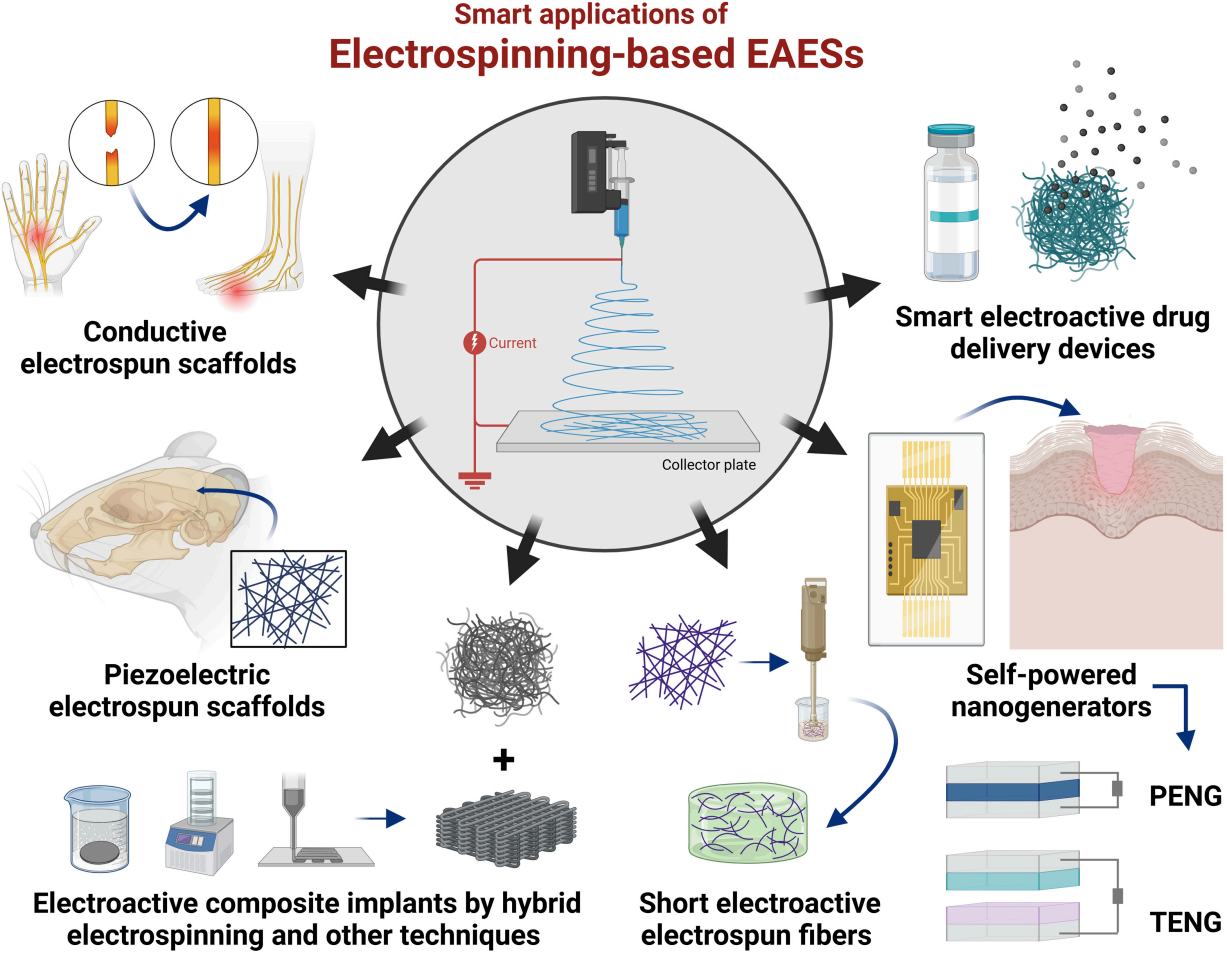
Smart applications of electrospinning-based electroactive electrospun scaffolds in tissue regeneration, including conductive/piezoelectric electrospun scaffolds, electroactive composite implants, self-powered nanogenerators, and smart electroactive drug delivery devices.

Electrospinning can be employed to construct diverse devices tailored to the electrical activity required by different tissues, thereby promoting tissue regeneration (Fig. 8). By selecting appropriate base electrospinning materials, electroactive materials, and electrospinning methods, conductive or piezoelectric EAESs can be prepared for direct use in distinctive tissue regeneration. EAESs can also be combined with scaffolds constructed by other techniques to form electroactive composite implants, overcoming the inherent limitations of 2D electrospun membranes. In addition, EAECs can be developed into self-powered nanogenerators that provide electric stimulations to target repair areas via wearable or implantable approaches, enhancing tissue regeneration. Moreover, leveraging the electrical response properties of certain electroactive materials, EAECs can serve as smart electroactive drug delivery devices for controlled drug release.

### Electroactive electrospun scaffolds

#### Conductive electrospun scaffolds

Electrospun conductive scaffolds play a pivotal role in promoting the regeneration of electrically excitable tissues including neural, cardiac, dermal, and skeletal muscle tissues. Functionalized electroactive fibrous scaffolds can integrate physical, chemical, electrical, and topographical properties to effectively recapitulate the native electrophysiological microenvironment, thereby supporting tissue regeneration under various pathological conditions.

**Fig. 9.**
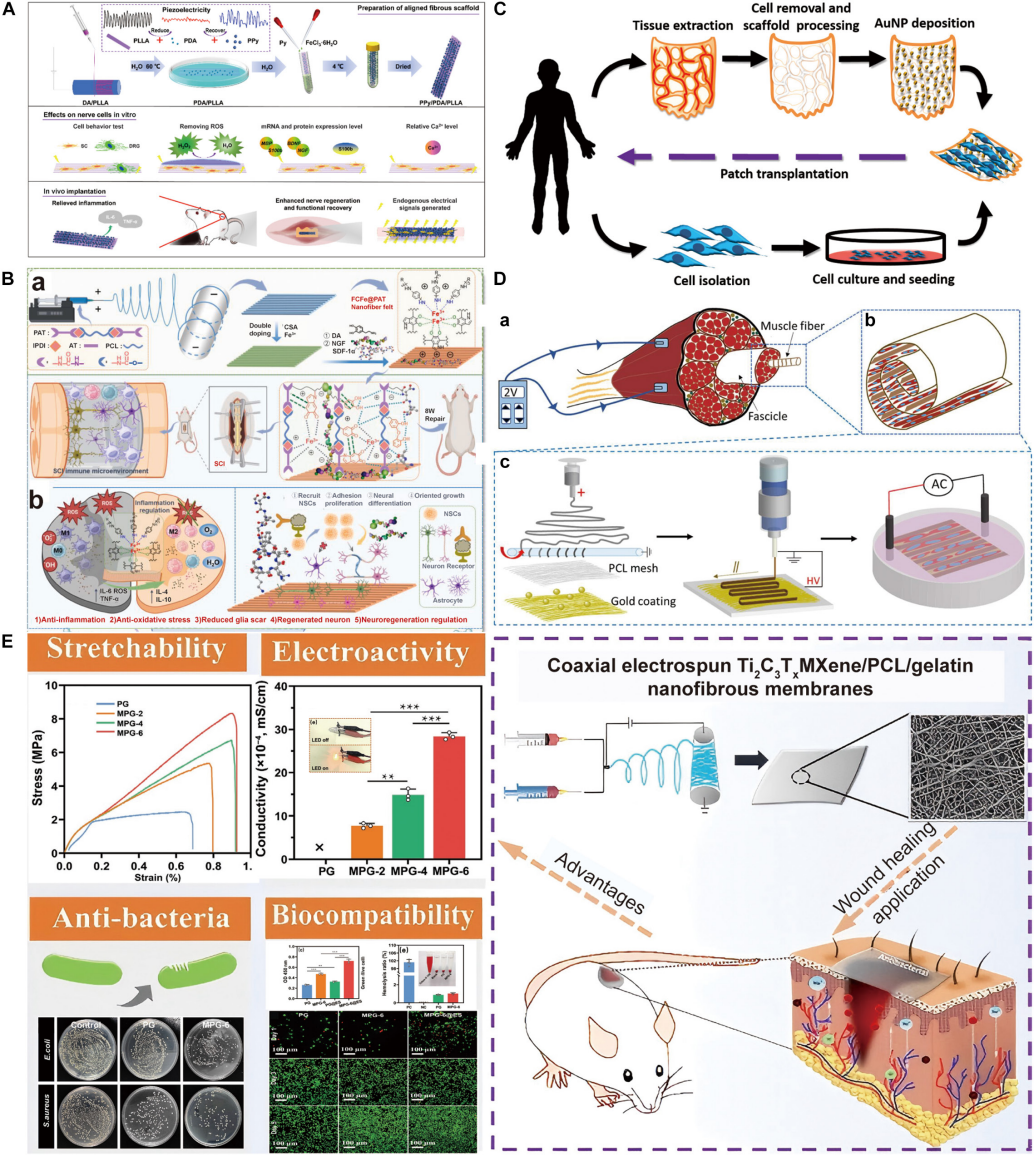
Applications of conductive electrospun scaffolds for tissue engineering. (A) Scheme of the study design. (a) Preparation of aligned PPy/PDA/PLLA electrospun nanofiber scaffolds, (b) effects of fiber scaffolds on neural cell growth in vitro, and (c) in vivo implantation and characterization of fiber conduits during peripheral nerve reconstruction in rat models. Reproduced with permission [220]. Copyright 2023, ACS Publications. (B) Schematic illustration of the fabrication and application of bioinspired conductive aligned nanofiber mats (FCFe@PAT). (a) Preparation of FCFe@PAT nanofiber mats, and (b) formation of multiple intermolecular and intramolecular FCFe@PAT forces on the nanofiber mats, and their immunomodulatory effects on spinal cord repair through anti-inflammatory actions, antioxidant stress reduction, glial scar suppression, neuronal regeneration, and neural regeneration regulation. Reproduced with permission [245]. Copyright 2025, Elsevier. (C) Schematic overview of the concept. Omentum tissue is isolated from patients and undergoes a rapid decellularization process to generate 3D scaffold biomaterials. Reproduced with permission [246]. Copyright 2014, ACS Publications. (D) (a) Schematic illustration of electrical stimulation on skeletal muscle using electrical pulses, (b) design of rolled scaffold with side-view configuration to mimic natural skeletal muscle tissue, and (c) fabrication diagram of 3D conductive scaffolds for electrical stimulation of muscle cells. Reproduced with permission [248]. Copyright 2021, Elsevier. (E) Schematic of the design of electroactive and antibacterial wound dressing based on Ti_3_C_2_T_x_ MXene/poly(ε-caprolactone)/gelatin coaxial electrospun nanofiber membranes. Reproduced with permission [249]. Copyright 2023, Springer Nature.

Electrospun conductive scaffolds have found extensive applications in neural regeneration, where scaffold conductivity and radial alignment emerge as 2 critical determinants of repair efficacy. For instance, Xiong et al. [220] developed aligned piezoelectric and conductive PPy/polydopamine (PDA)/PLLA electrospun fiber scaffolds for neural regeneration (Fig. 9A). PPy was uniformly distributed on aligned PLLA fibers via in situ polymerization, while dopamine was employed to enhance PPy-PLLA interfacial bonding and improve composite hydrophilicity—providing a novel approach for artificial nerve scaffold optimization. Building upon this, Pi’s team further functionalized scaffolds by incorporating carboxylated multiwalled CNTs (MWCNT-COOH) into PCL matrices while loading brain-derived neurotrophic factor (BDNF). This design not only maintained conductivity but also substantially activated SC myelination gene expression [240]. However, the introduction of carbon nanomaterials requires careful consideration as randomly dispersed carbon nanomaterials tend to compromise conductivity [241]. To address this, carbon-based materials are typically modified with redox-active, carboxyl, or PDA functional groups. GO and rGO represent the most extensively studied covalent functionalization forms [242]. Zhou et al. [243] developed GO-incorporated PCL nanofiber scaffolds with aligned microstructures and electroactivity for neural reconstruction. Results revealed that 0.5% GO/PCL nanocomposite scaffolds achieved superior conductivity (0.09 ± 0.01 S/cm). Beyond conductivity and alignment, scaffold hydrophilicity must be optimized given the inherent hydrophobicity of conductive polymers and carbon nanomaterials. Imani et al. [244] engineered a neural tissue engineering scaffold combining PLA nanofibers, gelatin, and PPy, merging the topographic advantages of electrospun nanofibers with the multifunctionality of gelatin–PPy composites. Through grafting varying pyrrole ratios onto gelatin chains followed by surface conjugation to PLA nanofibers, the resulting conductive scaffolds exhibited excellent hydrophilicity, conductivity, and cell-adhesion properties.

Compared to peripheral nerve injuries, spinal cord injury involves complex interactions among neurons, glial cells (e.g., astrocytes and oligodendrocytes), vascular systems, and immune microenvironments. Zhang et al. [245] synthesized stretchable conductive polyurethane copolymers via addition polymerization, fabricating nanofiber mats through electrospinning with topographic guidance (Fig. 9B). Subsequent doping with camphorsulfonic acid and ferric chloride enhanced conductivity while conferring antibacterial and hemostatic properties. A PDA coating deposited on nanofiber surfaces with adherent NGF and SDF-1α further improved immune microenvironment responsiveness, creating a biomimetic conductive aligned nanofiber mat capable of simultaneous inflammation suppression and neural regeneration.

Electrospun conductive fibers exhibit unique advantages in cardiac regeneration by simultaneously mimicking the anisotropic structure and electrical conduction system of myocardial tissue, and they can restore both electrical signal conduction and tissue structure, substantially improving cardiomyocyte maturation and contraction synchrony. Liu et al. [127] developed CNT-incorporated aligned PELA fibers for cardiac tissue engineering using blend and coaxial electrospinning. At 3% CNT loading, these fibers achieved conductivities of ≈30.7 mS/cm (blend) and 26.1 mS/cm (coaxial), with blended fibers demonstrating superior conductivity. Conductive nanoparticles outperform carbon nanomaterials in conductivity enhancement. Shevach et al. [246] deposited conductive AuNPs on PCL–gelatin hybrid nanofiber scaffolds, substantially increasing cardiomyocyte aspect ratios and enhancing cardiac sarcomeric α-actinin expression (Fig. 9C). Diedkova et al. [247] pioneered a novel cardiac patch containing MXenes, and oxygen plasma treatment enhanced MXene infiltration into PCL electrospun membranes, improving cell adhesion. The PCL-MXene membranes exhibited remarkable conductivity enhancement (from 5.22 to 326.33 mS/m) alongside excellent cellular responses [247].

Electrospun conductive scaffolds have expanded from neural applications to skeletal muscle regeneration and skin wound healing. In dermal repair, conductive scaffolds not only promote keratinocyte migration and proliferation via electric stimulations but also regulate growth factor secretion through their conductive networks, accelerating wound closure. Concurrently, their inherent antibacterial properties prevent wound infection, creating a favorable regenerative microenvironment. For skeletal muscle regeneration, anisotropic conductive scaffolds mimic native myofiber alignment and electrophysiology, precisely guiding myoblast orientation and fusion to form functional myotubes. Zhang et al. [248] developed conductive PCL/Au scaffolds with anisotropic micro/nanostructures using combined electrospinning, surface coating, and melt electrospinning (Fig. 9D). Cell experiment results show that enhanced electric stimulation of PCL/Au scaffolds can promote the expression of sarcomeric α-actin during the development of sarcomere in differentiated myotubule, thereby promoting myotubule formation and skeletal muscle regeneration [248]. In another study, Xu et al. [249] leveraged Ti_3_C_2_T*_x_* MXene’s superior conductivity and antibacterial activity to fabricate core–shell structured nanofiber membranes via coaxial electrospinning (PCL/gelatin shell with MXene core) (Fig. 9E). Minimal MXene incorporation (0.1 wt%) yielded membranes with excellent conductivity (overcoming low conductivity issues in blended fibers) while maintaining antibacterial efficacy. Similarly, Xiong et al. [250] first prepared poly(L-lactide) nanofibers via electrospinning, then successfully constructed a composite dressing with multilayer core–shell structure through surface modification using in situ polymerization. Through the synergistic modification of PDA and PPy, the material demonstrated substantially improved surface hydrophilicity and enhanced electrical conductivity. Experimental results showed that this composite nanofiber exhibited excellent photothermal antibacterial effects, promoted wound hemostasis, and demonstrated good free radical scavenging capability. These characteristics make it highly valuable for clinical wound repair applications.

#### Piezoelectric electrospun scaffolds

Unlike conductive materials, piezoelectric materials utilize their intrinsic piezoelectric properties without requiring external electrical signals. Electrospun conductive scaffolds play a crucial role in promoting regeneration of electroactive tissues including nerves, osteochondral structures, and skin.

**Fig. 10.**
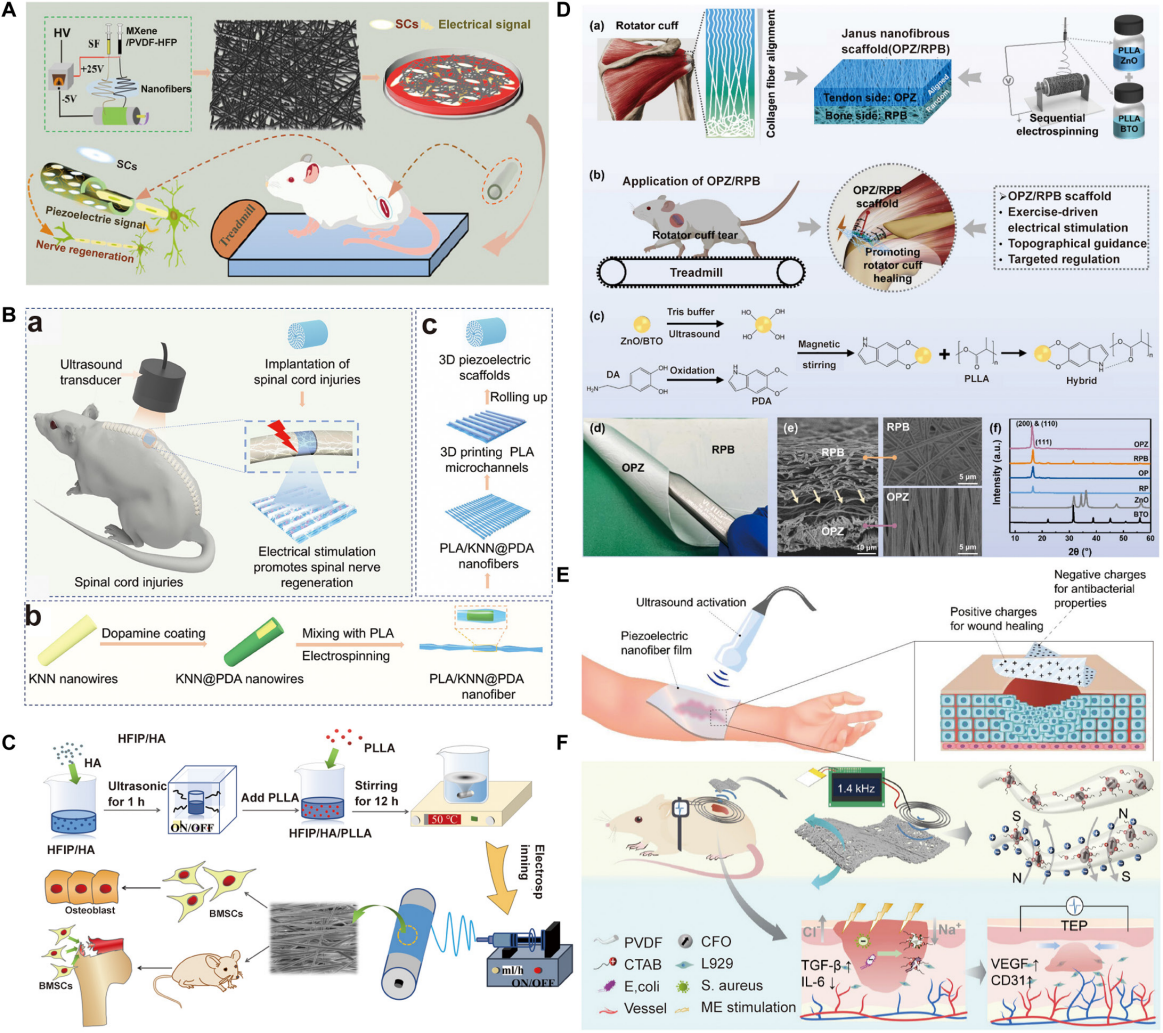
Applications of piezoelectric electrospun scaffolds for tissue engineering. (A) Schematic diagram of electrospun piezoelectric scaffolds under external mechanical stimulation promote peripheral nerve injury regeneration. Reproduced with permission [251]. Copyright 2023, ACS Publications. (B) Schematic diagram of biodegradable 3D piezoelectric scaffolds promoting spinal cord repair. (a) Biodegradable high-performance 3D piezoelectric scaffolds can promote spinal cord repair, (b) preparation of PLA/KNN@PDA nanofibers, and (c) fabrication process of 3D piezoelectric PLA/KNN@PDA nanofiber scaffolds. Reproduced with permission [253]. Copyright 2022, ACS Publications. (C) Schematic diagram of preparation process of PLLA-HA nanofibrous membrane. Reproduced with permission [254]. Copyright 2022, Elsevier. (D) Fabrication and characterization of Janus nanofiber scaffolds. (a) Schematic diagram of scaffold fabrication; (b) potential mechanism of scaffold-mediated synchronous tendon–bone healing; (c) schematic of possible interfacial interactions among ZnO/BaTiO_3_ nanoparticles, PDA, and PLLA; (d) optical image of OPZ/RPB scaffold; (e) SEM images; and (f) XRD analysis. Reproduced with permission [256]. Copyright 2024, Elsevier. (E) The piezoelectric PLLA nanofiber mat serves as an externally controlled electrical stimulator to promote wound healing and antibacterial effects. Reproduced with permission [258]. Copyright 2023, Elsevier. (F) Schematic of electrospun CoFe2O4@CTAB/PVDF dressing enables wearable magnetoelectric stimulation for chronic wound healing. Reproduced with permission [259]. Copyright 2024, ACS Publications.

When fabricating piezoelectric scaffolds, PVDF has been extensively studied due to its excellent flexibility and high piezoelectric performance. However, PVDF’s poor biodegradability has limited its further development. Therefore, new strategies are still needed to further improve biodegradability and biocompatibility. SF possesses not only tunable water solubility and excellent biocompatibility and biodegradability, but also shear and longitudinal piezoelectric properties, making it ideal for constructing implantable piezoelectric scaffolds. Based on this, Zhang et al. [251] developed an SF/PVDF-HFP/MXene piezoelectric composite scaffold using electrospinning technology (Fig. 10A). The incorporation of MXene enhanced the scaffold’s piezoelectric performance (with output voltage reaching up to 100 mV), mechanical properties, and antibacterial activity. The addition of SF improved the biocompatibility of the piezoelectric scaffold. Cell experiments demonstrated that piezoelectric stimulation under external ultrasound promoted the growth and proliferation of SCs cultured on this electrospun scaffold. In addition, Zhen et al. [252] developed dry-spun piezoelectric fibers based on SF. By regulating β-sheet content through post-processing, they enhanced piezoelectric performance. The resulting SF-based piezoelectric generator achieved a maximum electrical output of 27 V, applicable for detecting human finger joint movements and promoting PC-12 cell growth, demonstrating great potential for neural repair applications.

Similar to conductive scaffolds, piezoelectric scaffolds can also facilitate spinal cord repair. Chen et al. [253] incorporated biodegradable high-performance potassium sodium niobate (KNN) piezoelectric material into PLA nanofibers, constructing a 3D biodegradable piezoelectric scaffold with ultrasound-driven wireless stimulation capability through electrospinning (Fig. 10B). Under appropriate ultrasound excitation, the 3D tissue scaffold made of piezoelectric composite nanofibers generated endogenous electrical signals that promoted neural stem cell differentiation and angiogenesis at the lesion site, accelerating motor function recovery and enhancing spinal cord repair.

Osteochondral tissue naturally contains abundant mechanical signals due to human movement requirements, making it particularly suitable for piezoelectric scaffold applications. When applying these scaffolds, special attention should be paid to piezoelectric potential, radial properties, biodegradability, and spatial structure. For example, Lv et al. [254] developed hydroxyapatite (HA)-incorporated aligned PLLA nanofiber membranes via electrospinning as cost-effective rotator cuff patches (Fig. 10C). The membranes showed excellent cytocompatibility with cells aligning along fiber direction. Furthermore, PLLA-HA nanofiber membranes increased alkaline phosphatase (ALP) expression in BMSCs, indicating enhanced osteogenic induction. To better enhance the radial synergistic electroactivity of scaffolds, Wang et al. [255] developed a piezoelectric aligned nanofiber scaffold composed of ZnO@PCL/PVDF using electrospinning. Under ultrasound control, its piezoelectric output could mimic physiological electrical signals of healthy bone tissue, providing antibacterial, immunomodulatory, and osteogenic effects by accurately replicating endogenous electrical microenvironments. Besides, Liu et al. [84] created biodegradable PLLA nanofiber scaffolds serving as battery-free electrical stimulators to promote chondrogenesis and cartilage regeneration. PLLA scaffolds generated controlled piezoelectric charges under mechanical loading, promoting extracellular protein adsorption, cell migration/recruitment, and endogenous TGF-β induction via Ca^2+^ signaling pathway, improving chondrogenesis both in vitro and in vivo [84].

Most aforementioned electroactive nanofibers have monolayer structures unable to simultaneously achieve multiple functions (e.g., biocompatibility, electrical properties, and topographic features). Zhang’s team innovatively developed Janus-structured piezoelectric nanofiber scaffolds by electrospinning back-to-back layers of PLLA/ZnO and PLLA/BTO, synergistically promoting tendon–bone interface tissue regeneration by mimicking natural collagen fiber alignment (Fig. 10D) [256]. Cell experiment results showed that the gene expression of osteogenesis-associated Runx2, Col I, and OCN were substantially increased after 14 days, and it may be correlated with the activation of voltage-gated calcium channels Role: Author, stretch-activated calcium channels, and mechanoreceptors such as integrins, thus inducing the downstream signaling pathways for osteogenic differentiation [256]. Although Janus fibers have multilayer structures, they remain essentially 2D piezoelectric scaffolds. Compared to traditional 2D scaffolds, 3D piezoelectric scaffolds more accurately simulate natural ECM structures through porous networks, providing cells with 3D electroactive growth environments that enable multidimensional coordination of cellular behaviors (e.g., migration and differentiation), offering regeneration pathways closer to physiological conditions for complex tissues (e.g., skin, muscle, and bone). Muenwacha et al. [257] fabricated piezoelectric 3D PVDF-HFP scaffolds for bone tissue engineering using wet electrospinning. Moreover, the 3D nanofiber architecture could generate more electrical signals than conventional 2D structures because the third dimension provides additional compression. Cell interactions with 3D nanofiber scaffolds were investigated, with in vitro results showing that NIH3T3 cells could attach and migrate within the 3D structure.

In skin regeneration, compared to conductive scaffolds, piezoelectric scaffolds generate dynamic electrical signals through mechanical stress to promote cell migration and collagen remodeling, making them more suitable for dynamic wound repair. For example, Das et al. [258] synthesized a novel biodegradable, multifunctional, and self-powered piezoelectric scaffold for skin regeneration (Fig. 10E). By collecting electrospun fibers at rotational speeds of 300 and 4,000 rpm, they enhanced the fiber alignment of the piezoelectric scaffold, thereby improving both its piezoelectric and mechanical properties. Compared with randomly aligned fibers, the oriented fibers facilitated better infiltration and proliferation of epithelial cells and fibroblasts, which promoted wound healing. In addition to dynamic wound healing applications, researchers are exploring its potential for chronic skin wound treatment. Ke et al. [259] developed a magnetoelectric dressing called CFO@CTAB/PVDF. Cetyltrimethylammonium bromide (CTAB) served as a dispersing surfactant for CFO, with its quaternary ammonium cations endowing the dressing with both antibacterial and hydrophilic properties (Fig. 10F). This magnetoelectric dressing utilizes a wearable electromagnetic induction device to establish a dynamic magnetic field that induces magnetostrictive deformation of CFO nanoparticles. Histological and molecular biological evaluations confirmed CTAB’s anti-inflammatory effects as well as the electric stimulation’s ability to accelerate collagen deposition and vascular formation.

To summarize, electrospun conductive scaffolds and piezoelectric scaffolds demonstrate unique advantages in tissue regeneration. Conductive scaffolds promote the regeneration of neural, cardiac, and skin tissues by mimicking native electrophysiological microenvironments, while piezoelectric scaffolds generate dynamic electrical signals through mechanical stress, making them more suitable for dynamic wound repair. Both types of scaffolds can enhance conductivity, biocompatibility, and functional integration through material modifications and structural design. Therefore, despite remaining challenges, electroactive scaffolds exhibit broad potential in tissue engineering applications

### Electroactive composite implants by hybrid electrospinning and other techniques

While EAESs showed great potential to promote tissue regeneration by their inherent electric stimulations, it is still difficult to fully stimulate 3D tissue microenvironment because conventional electrospinning generally fabricates 2D membranes. Thus, electrospinning can be combined with other scaffold fabrication techniques to construct electroactive composite implants for tissue engineering, and there are mainly 2 types of composite implants including hierarchical composite implants containing EAESs and short electroactive electrospun fibers containing composite implants.

#### Hierarchical composite implants containing EAESs

**Fig. 11.**
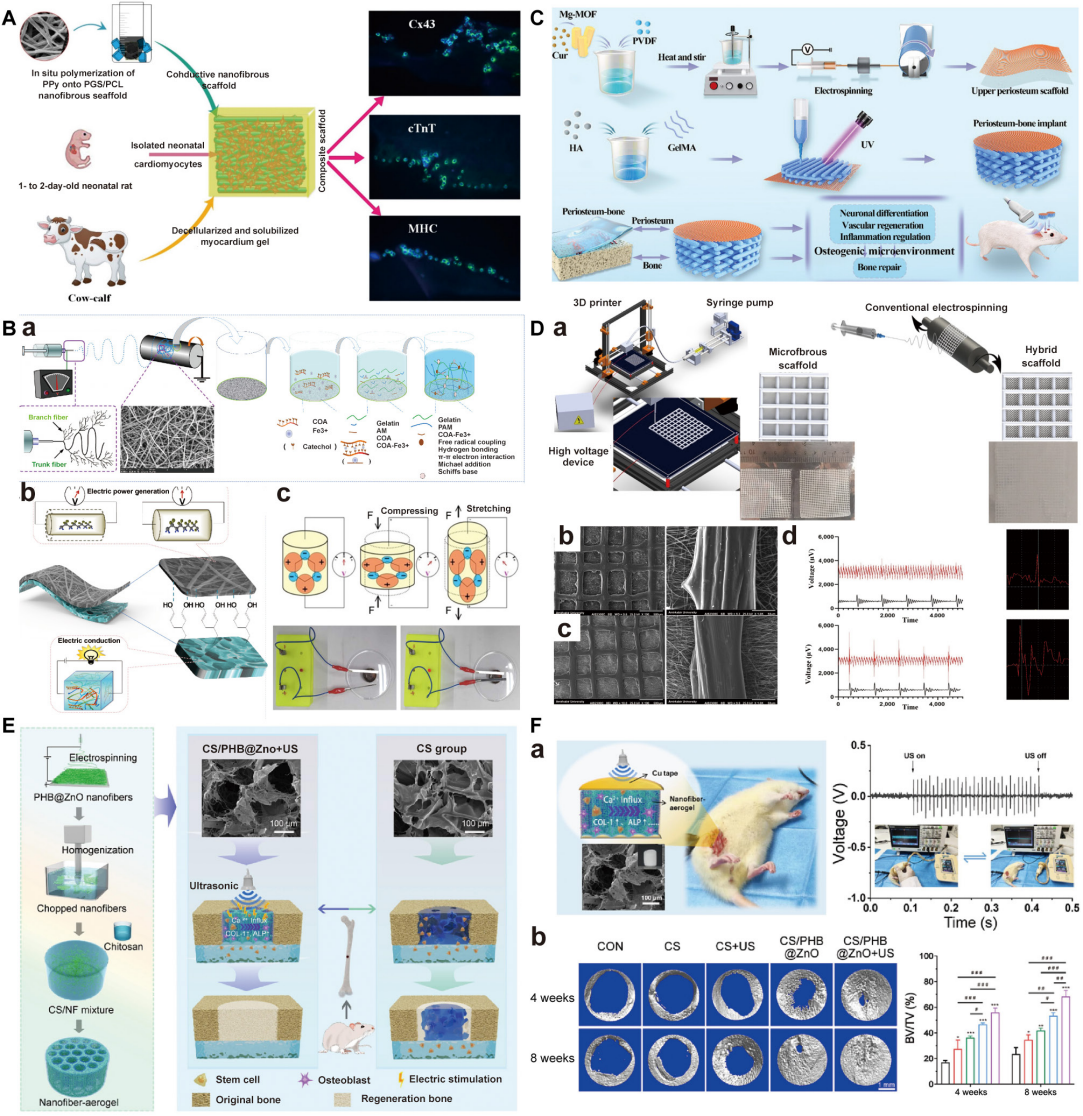
Applications of composite implants containing electroactive electrospun scaffolds and short electroactive electrospun fibers. (A) Schematic illustration of conductive electrospun nanofibers in the matrix of hydrogel for myocardial infarction therapy. Reproduced with permission [261]. Copyright 2024, ACS Publications. (B) Schematic illustration of piezoelectric electrospun nanofibers on a conductive adhesive hydrogel. (a) Fabrication process, (b) piezoelectric properties and mussel-inspired adhesion mechanism, and (c) self-powered electric signals to light the bulb. Reproduced with permission [262]. Copyright 2023, ACS Publications. (C) Schematic illustration of a bilayer periosteum–bone biomimetic scaffold fabricated by electrospinning and 3D printing for bone regeneration. Reproduced with permission [265]. Copyright 2025, Wiley-VCH. (D) Composite implants fabricated by sequential near-field electrospinning and conventional electrospinning. (a) Schematic illustration of fabrication procedure, (b and c) representative SEM images of composite implants containing random or aligned piezoelectric electrospun nanofibers, and (d) piezoelectric properties of random or aligned piezoelectric electrospun nanofibers. Reproduced with permission [163]. Copyright 2025, Elsevier. (E) Schematic illustration of short piezoelectric electrospun nanofibers in aerogel scaffolds. Reproduced with permission [77]. Copyright 2023, Elsevier. (F) Application of piezoelectric nanofiber-aerogel scaffolds for bone regeneration. (a) Generated piezoelectric potential under the stimulation of ultrasound, and (b) representative micro-CT results and quantitative BV/TV values. Reproduced with permission [77]. Copyright 2023, Elsevier.

EAESs could be combined with scaffolds fabricated by other techniques to form hierarchical composite implants. Hydrogels are 3D network materials formed by the physical or chemical crosslinking of polymers or supramolecules, and they possess high water content and can provide a 3D ECM-mimicking microenvironment [260]. Thus, EAESs can be combined with hydrogels to fabricate 3D composite implants for tissue engineering. On the one hand, EAESs can be introduced into the hydrogel matrix. As an example, Fakhrali et al. [261] first coated conductive PPy on the surface of PGS/PCL electrospun fibers by in situ polymerization, fabricating a conductive electrospun scaffold with an electrical conductivity 0.00108 S cm^–1^. The conductive scaffold was then introduced to a decellularized myocardium gel to fabricate a composite implant for myocardial infarction therapy (Fig. 11A), and the hydrogel could provide a 3D cellular microenvironment with favorable physicochemical characteristics [261]. The results showed that conductive PPy and the hydrogel could synergistically promote cell infiltration and the expression of myocardial maturation markers including Cx43, MHC, and cTnT proteins [261]. On the other hand, EAESs can be loaded on the hydrogels to form hierarchical composite scaffolds. For example, Chen et al. [262] fabricated a 2-layered biomimetic flexible self-powered electrical-stimulator-based wound dressing by integrating a tree-like bionic piezoelectric electrospun layer with a conductive adhesive hydrogel containing Fe^3+^ as a carrier of electron conduction and catechol group for adhesion (Fig. 11B, a and b). The composite could transfer mechanical stimulation to electric signals (Fig. 11B, c), which further promoted cell proliferation and wound healing [262]. In another study, aligned piezoelectric electrospun nanofibers were combined with a thermoresponsive hydrogel containing therapeutic drugs for peripheral nerve regeneration [263]. The aligned piezoelectric electrospun fibers not only guided the directional growth of neurons but also enabled wireless stimulation under ultrasound. Simultaneously, the thermosensitive hydrogel contracted due to the thermal effect of ultrasound, triggering drug release. Thus, the topological structure, electrical signals, and bioactive factors collectively promote the functional recovery and nerve axonal regeneration facilitated by this composite scaffold [263].

Additive manufacturing, generally known as 3D printing, is a technique that constructs 3D objects by adding material layer by layer, and it can be used to fabricate customed scaffolds with controlled structure, porosity, and pore size for tissue engineering [264]. Electrospinning can be combined with 3D printing to develop composite implants comprising EAESs and printed scaffolds. For example, Yue et al. [265] fabricated a bilayer periosteum–bone biomimetic scaffold that was composed of an upper piezoelectric electrospun membrane and a lower printed scaffold (Fig. 11C). The upper layer was fabricated by electrospinning with PVDF and curcumin-loaded magnesium metal–organic framework to provide piezoelectric signals and the released curcumin and magnesium, and the lower scaffold was constructed with 3D printing to fill the bone defects and facilitate osteogenesis of BMSCs [265]. The results showed that the composite implant can accelerate bone regeneration by neurogenesis, angiogenesis, osteogenesis, and immune modulation, mainly by JAK-STAT, PI3K-Akt, HIF-1α, and TGF-β signaling pathways [265].

In addition to conventional additive manufacturing, NFES and MEW integrated from both electrospinning and 3D printing have gained great attention because they show advantages including a small fiber diameter, a microfibrous architecture, a controlled pore size, and an adjusted scaffold structure. In one study, Khodabandeh et al. [163] first utilized NFES to fabricate a microfibrous PCL/HA scaffold to provide large pores (>300 μm) for bone regeneration, and then used electrospinning fabricated aligned or random electrospun PLLA piezoelectric nanofibers to enhance cell adhesion and provide electric stimulation (Fig. 11D, a to c). The results showed that aligned PLLA nanofibers showed enhanced piezoelectric properties (Fig. 11D, d), and the improved electric stimulation could further improve osteoinductive properties [163]. In another study, researchers fabricated a tri-layer NGC for peripheral nerve regeneration by the combination of electrospinning and MEW [266]. Electrospinning was used to fabricate the outer PCL/collagen nanofibers to permit high permeability for the exchange of nutrition and waste while blocking infiltration of fibrous scar tissue [266]. MEW was used to fabricate the internal layer composed of longitudinal PCL microfibers to anisotropically guide neuronal growth while rapidly recruiting vascular cells and macrophages, and the intermediate layer consisting of rGO/PCL microfibers for mechanical stability and the transmission of electric signals [266]. The composite NGC facilitated SC elongation/proliferation and PC12 cell neurite outgrowth in vitro, while also stimulating neovascularization and peripheral nerve regeneration in vivo [266].

Thus, hierarchical composite implants fabricated by the combination of electrospinning and other scaffold fabrication techniques can not only mimic the 3D ECM microenvironment but also replicate the layered structures of tissues or tissue interfaces. Compared to the sole application of EAESs, electroactive composite implants offer unique advantages from other scaffolds such as structural support and enhanced cell infiltration, demonstrating great potential for tissue engineering.

#### Short electroactive electrospun fibers contained composite implants

Beyond directly combining EAESs with scaffolds fabricated by other techniques, EAESs can be cut into short electrospun fibers and then introduced into other biomaterials to fabricate composite implants for tissue regeneration. For example, Chen et al. [77] first prepared short piezoelectric PHB@ZnO short fibers by electrospinning coupled with subsequent homogenization and chopping, and then incorporated them in chitosan matrix to fabricate nanofiber-aerogel scaffolds for bone regeneration by the vacuum freeze-drying method (Fig. 11E). Under ultrasound stimulation, the composite implant could promote electric signals to promote the osteogenic differentiation of BMSCs by improving intracellular Ca^2+^ levels and subsequent CaM/CaN/NFAT signaling pathway and apparently promote bone defect healing in vivo (Fig. 11F) [77]. In another study, piezoelectric PLLA electrospun fibers were cut into short fibers by cryo-section, and then they were loaded in a collagen hydrogel for osteoarthritis treatment [83]. The results showed that the piezoelectric composite hydrogels could promote stem cells to secrete TGF-β1 and then enhance chondrogenesis and subchondral bone formation [83]. In addition to hydrogels, short electroactive electrospun fibers showed promise to be added in other biomaterials such as 3D printing inks for customized scaffolds for tissue engineering.

### Self-powered nanogenerators

**Fig. 12.**
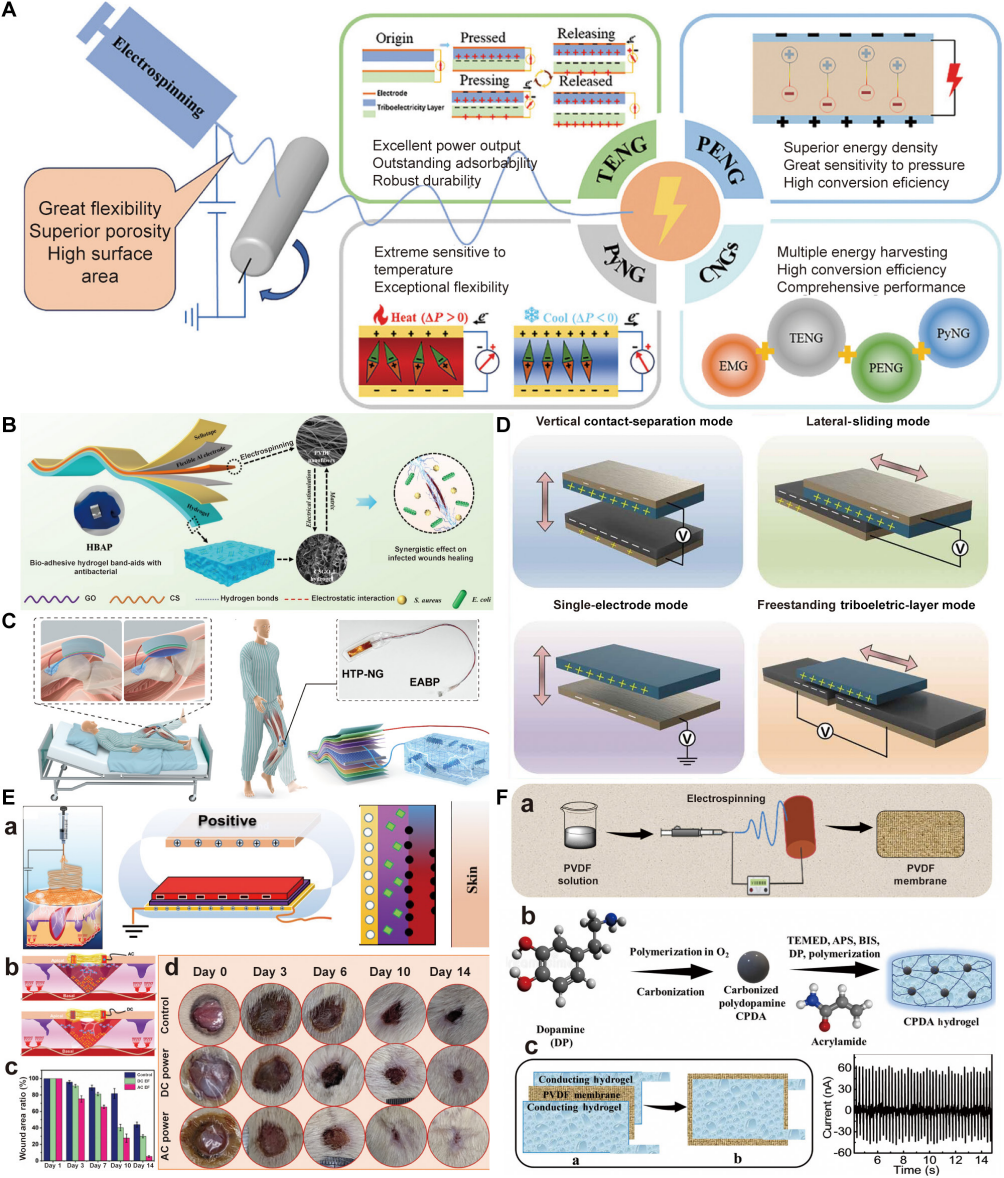
Applications of self-powered nanogenerators fabricated by electrospinning. (A) Schematic illustration of electrospinning to fabricate distinctive self-powered nanogenerators. Reproduced with permission [267]. Copyright 2025, Wiley-VCH. (B) Schematic illustration of a piezoelectric nanogenerator coupled with conductive hydrogel for infected wound healing. Reproduced with permission [269]. Copyright 2024, Elsevier. (C) Schematic illustration of a piezoelectric nanogenerator to be implanted subcutaneously for bone regeneration by rehabilitation exercise. Reproduced with permission [79]. Copyright 2024, AAAS. (D) Four operating modes of triboelectric nanogenerator. Reproduced with permission [270]. Copyright 2025, Elsevier. (E) Application of a single electrode mode triboelectric nanogenerator for wound healing. (a) Schematic illustration of the fabrication and structure of the triboelectric nanogenerator, (b) application models of the triboelectric nanogenerator, (c) quantitative wound area ratio, and (d) representative images of wounded skin at different time after treatment. Reproduced with permission [236]. Copyright 2024, Wiley-VCH. (F) Application of a piezo-driven triboelectric nanogenerator as wound dressing. (a) Fabrication of a piezoelectric electrospun PVDF membrane as an active layer for the nanogenerator, (b) fabrication of a conductive hydrogel as an electrode for the nanogenerator, and (c) the structure of the nanogenerator and its electric signals. Reproduced with permission [271]. Copyright 2022, Elsevier.

Self-powered nanogenerators are micro-nano scale energy conversion devices that transform mechanical, thermal, or biological energy from the environment into electrical energy through specific physical mechanisms, and electrospinning showed great promise to fabricate smart devices due to its great flexibility, superior porosity, high surface area, and versatility (Fig. 12A) [267]. According to the type of energy collected, there are various self-powered nanogenerators including piezoelectric nanogenerators (PENGs), triboelectric nanogenerators (TENGs), pyroelectric nanogenerators, electromagnetic nanogenerators, thermoelectric nanogenerators, and coupled nano-generators [267]. In the field of tissue engineering and regenerative medicine, PENGs and TENGs are the most widely applied nanogenerators because they can be worn or implanted, converting the body’s own mechanical energy into electrical signals to promote tissue regeneration.

Compared to conventional electroactive scaffolds in tissue engineering, nanogenerators incorporate electrode design, whose core function is to efficiently collect internal bound charges and convert them into free charges and current that can be outputted. This current is then delivered through a closed circuit to the target area to promote tissue repair. In contrast, piezoelectric scaffolds lie in the localized electrical signals generated on the material surface through its inherent piezoelectric properties to exert direct electrical effects on topical tissue regeneration, while conductive scaffolds typically conduct endogenously generated electrical signals in electroactive tissues to restore functionality in damaged areas. Both piezoelectric and conductive scaffolds do not require specially designed electrodes to collect and output large currents. The differences between self-powered nanogenerators and electroactive scaffolds (piezoelectric or conductive) are summarized in Table 5.

**Table 5.** The differences between self-powered nanogenerators and electroactive scaffolds

Aspect	Self-powered nanogenerators	Piezoelectric scaffolds	Conductive scaffolds
Core purpose	Harvest mechanical, thermal, or biological energy → Convert to electricity	Provide structural support + Utilize self-generated electrical signals to stimulate cells	Provide structural support + Transmit external/internal electrical signals
Energy source	Endogenous mechanical energy (e.g., rehabilitation exercise, heartbeat, and respiration)	Endogenous mechanical energy (physiological micro-stresses)	Requires external power source (unless integrated with generators)
Working mechanism	Piezo/tribo/pyroelectric effect → Output current/voltage	Piezoelectric effect → Localized surface potential → Direct cell stimulation	Conductive network → Transmits external stimuli or conducts cellular electrical signals
Key components	Essential electrodes + self-powered electroactive layer + encapsulation	Piezoelectric materials, no electrodes needed	Conductive materials, no electrodes needed
Output form	Usable electrical energy (powers external devices)	Localized electric field/potential (acts on cellular microenvironment)	Electrical signal transmission channel (conducts external current)
Cell interaction	Indirect (via powered devices)	Direct (surface electric field regulates cell behavior)	Direct (conductive interface delivers signals to cells)
Primary advantages	Self-sustaining, battery-free, continuous power supply	Self-powered stimulation, biomimetic microenvironment, real-time responsiveness	High conductivity, tunable stimulation parameters, easy functionalization
Key limitations	Limited power output, long-term stability challenges	Weak signal intensity, difficult to control precisely	Requires external power, risk of electrochemical side reactions
Power types	Self-powered	Self-powered	Electric signals from electroactive tissue
Application modes	Wearable, externally fixed, or implanted	Implanted	Implanted

#### Piezoelectric nanogenerators

Unlike piezoelectric EAESs, PENGs incorporate electrodes that convert immobile polarized charges within the piezoelectric material into mobile free charges on the electrodes, and then these charges are delivered to targeted therapeutic zones through a closed circuit. Electrospinning-based PENGs can function as an external device for wound healing to convert mechanical stimuli into electrical signals for tissue regeneration [268]. For example, Lu et al. [269] first used electrospinning to fabricate a piezoelectric PVDF membrane and then pasted 2 conductive adhesive Al electrodes on the top and bottom surface of the PVDF layer. Then, Sellotape was used to wrap the Al electrodes and PVDF membrane edges while exposing the terminal ends of the Al electrodes to make contact with the peripheral region of the conductive hydrogel, thereby constructing a composite dressing with upper PENG to produce electric signals and upper conductive hydrogels to transfer electric signals to promote the healing of infected wounds (Fig. 12B) [269]. Moreover, when PENG is used to promote deep tissue regeneration such as bone, it can be implanted subcutaneously through small incisions and connected to conductive scaffolds in the defects through electrodes or wires, promoting tissue regeneration based on rehabilitation training (Fig. 12C) [79]. Further mechanism studies revealed that electric stimulation by PENG can improve the expression of the mechanosensitive proteins of PIEZO1 and PIEZO2 and promote Ca^2+^ import, and then induce the osteogenic differentiation of stem cells by PI3K/Akt, WNT, and MAPK signaling pathways [79]. However, current implantable PENG requires secondary surgical removal due to nondegradability, causing secondary trauma to patients. Thus, PENG is currently more suitable for repairing superficial tissues such as wound, while developing fully biodegradable PENG represents a promising research direction to advance their implantable applications.

#### Triboelectric nanogenerators

TENGs can convert mechanical energy to electricity on the basis of triboelectrification and electrostatic induction, and there are mainly 4 operating modes including vertical contact-separation mode, lateral sliding mode, single-electrode mode, and freestanding triboelectric-layer mode (Fig. 12D) [270]. Recently, electrospinning-based TENGs have been used for tissue repair. For example, Venkatesan et al. [236] sequentially used electrospinning to fabricate PVDF/polyurethane electrospun membrane as a tribo-active layer and TiO_2_-MXene/polystyrene electrospun membrane as a charge trapping interlayer on the bottom AgNP-coated styrene butadiene stretchable electrode, thus fabricating a single-electrode mode TENG for wound healing (Fig. 12E, a). Upon contact separation, the TENG produced the electric signals in the form of AC, and it could also generate DC when connected with a 4-wave bridge rectifier [236]. Two modes of electric signals were tested in vivo with TENG covering the wound surface, and the results showed that the therapeutic efficacy of the AC-based TENG was better than that of the DC-based TENG (Fig. 12E, b to d) [236]. In another study, a vertical contact-separation mode TENG comprising an electrospun PHB membrane (positive tribomaterial) and an expanded polytetrafluoroethylene membrane (negative tribomaterial) was fabricated for self-powered and sensing vascular grafts, and the results showed that the TENG could promote the growth of HUVECs and monitor hemodynamic conditions [271]. Moreover, researchers have developed a piezo-driven TENG as wound dressing by inserting a piezoelectric electrospun PVDF membrane (active layer) into conductive hydrogels (electrodes) (Fig. 12F, a and b). Upon external mechanical pressure, piezoelectric voltage was formed between 2 electrodes, inducing short circuit current within the dressing to promote wound healing (Fig. 12F, c) [271]. Therefore, various TENGs show great potential to generate electric signals to promote superficial tissue regeneration, and further studies should pay attention to the device development for deep tissue engineering.

### Smart electroactive drug delivery devices

**Fig. 13.**
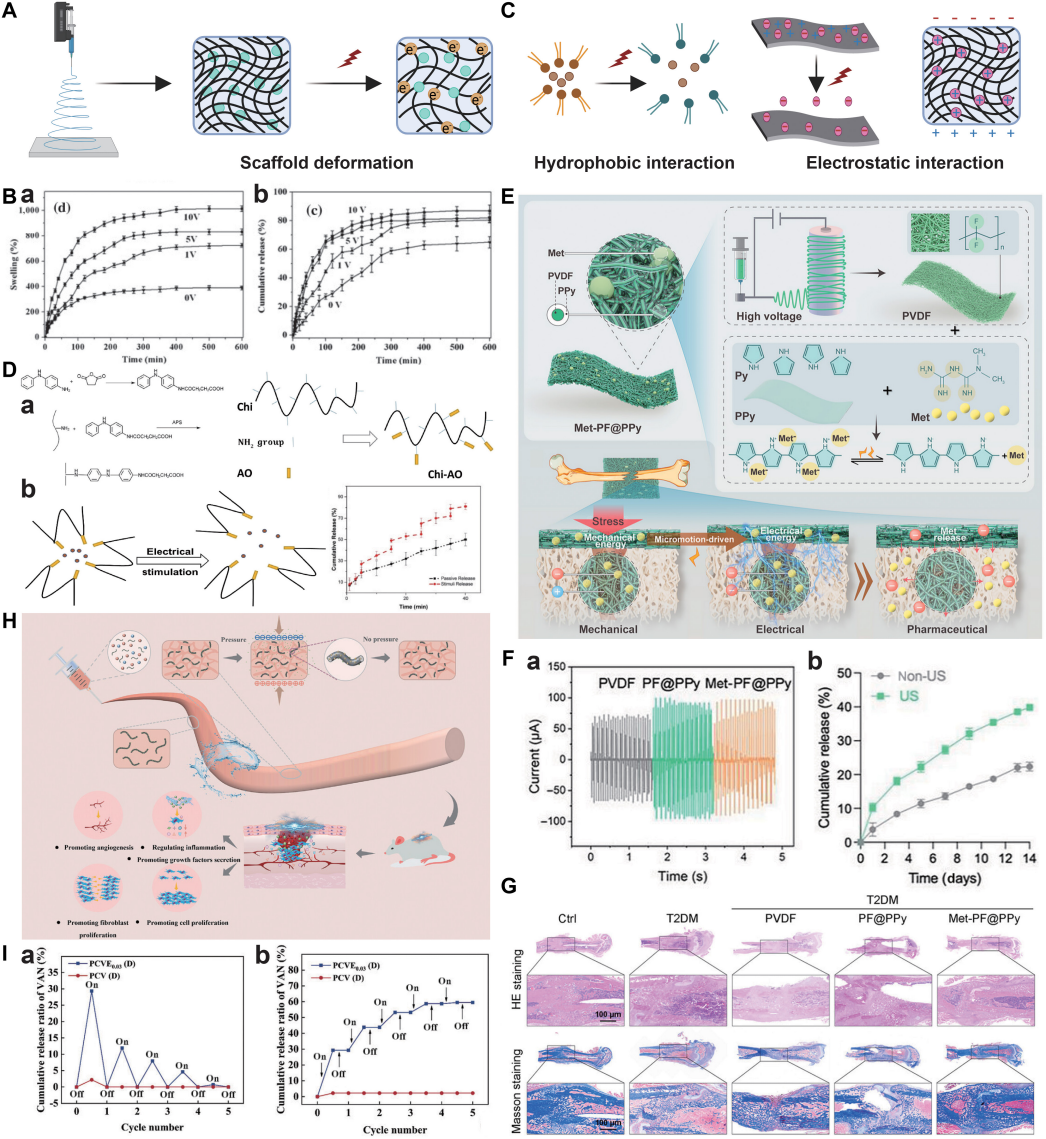
Applications of electroactive electrospun scaffolds as smart electroactive drug delivery devices. (A) The swelling, shrinking, or bending of the scaffold due to redox reactions and possible ionization initiated by exogenous electrical stimulations. (B) Electro-responsive release due to scaffold swelling. (a) The change of swelling under different electric voltages, and (b) cumulative release of the drug under different electric voltages. Reproduced with permission [273]. Copyright 2011, Elsevier. (C) Electric stimulations to regulate the noncovalent interactions between the drug and its carriers. (D) Electro-responsive release based on the hydrophobic interaction between hydrophobic drug and conductive chitosan-aniline oligomer. (a) Synthesis process of conductive chitosan-aniline oligomer, and (b) mechanism of electro-responsive release and stimulated drug release profile. Reproduced with permission [274]. Copyright 2020, Elsevier. (E) Schematic illustration of preparing of Met-PVDF@PPy through electrospinning for diabetic bone regeneration. Reproduced with permission [275]. Copyright 2025, Wiley-VCH. (F) (a) The output voltage of PVDF, PF@PPy, and Met-PVDF@PPy under the piezoelectric effect caused by low-intensity pulsed ultrasound stimulation, and (b) release kinetics of Met under piezoelectric effect. Reproduced with permission [275]. Copyright 2025, Wiley-VCH. (G) In vivo bone regeneration evaluated by HE staining and Masson staining. Reproduced with permission [275]. Copyright 2025, Wiley-VCH. (H) Schematic illustration of preparing and applying self-powered wound dressing based on “Lock-ON/OFF”. Reproduced with permission [276]. Copyright 2024, Wiley-VCH. (I) Electro-responsive release profiles. (a) The incremental release ratio, and (b) the cumulative release ratio under the emotional process. Reproduced with permission [276]. Copyright 2024, Wiley-VCH.

Smart stimuli-responsive delivery devices can achieve on-demand drug release at the targeted region and reduce the side effects from high doses, showing great potential for tissue engineering [272]. Exogenous electric stimulations can act on conductive EAESs to induce the electro-responsiveness of therapeutic agents for tissue regeneration. On the one hand, conductive EAECs may undergo deformation such as swelling, shrinking, or bending due to redox reactions and possible ionization initiated by exogenous electric stimulations, thus inducing the release of therapeutic cargos (Fig. 13A). For example, Yun et al. [273] modified hydrophobic MWCNTs by oxyfluorination to improve dispersion and then incorporated them into PVA/PAA electrospun scaffolds to improve electroconductivity. After an exogenous electric field was applied, the carboxyl groups (-COOH) on the polymer were protonated, generating -COO^-^ and H^+^. This leads to enhanced electrostatic repulsion, causing the polymer chains to extend and the swelling ratio to increase, thereby mediating the accelerated release of the internally loaded ketoprofen (Fig. 13B) [273]. On the other hand, exogenous electric stimulations could regulate the noncovalent interactions between the drug and the carrier, thereby promoting drug release (Fig. 13C). For example, GO was introduced to electrospun scaffolds containing quercetin to improve electroconductivity, and ion flow may disrupt interactions between scaffolds and quercetin under an exogenous electrical stimulus, thereby triggering the release of quercetin. In another study, Bagheri et al. [274] synthesized conductive chitosan by grafting aniline oligomers (Fig. 13D, a), followed by electrospinning the polymer solution with PVA to produce conductive nanofiber scaffolds. In the nonstimulated state, the hydrophobic domains of oligoaniline bind hydrophobic dexamethasone through noncovalent interactions, while the hydrophilicity of the aniline oligomer increases upon electric stimulation to facilitate the release of the hydrophobic drug (Fig. 13D, b).

Exogenous electric stimulations, however, present safety concerns and suboptimal therapeutic timeliness, limiting their clinical translation. In contrast, piezoelectric materials can transform localized mechanical energy into electrical energy, providing a self-powered solution for dynamic electric stimulation without external power requirements. Therefore, piezoelectric materials can be combined with conductive materials to fabricate smart electroactive drug delivery devices on the basis of their self-powered electric stimulations. For example, electrospun PVDF fibrous membranes functionalized with a metformin (Met)-loaded PPy coating have been developed (Fig. 13E) [275]. PPy is in an oxidized state (positively charged) during the preparation of the stent; thus, it could adsorb the anionic drug Met to maintain charge equilibrium and form a stable structure. Under ultrasound stimulation, the EAES could convert the mechanical energy into electrical signals, activating the electrochemical reduction of PPy (electric neutral) to enable the electro-responsive release of the anionic drug Met (Fig. 13F) [275]. The results showed that the scaffold could synthetically combine electric stimulation and Met to promote the osteogenic differentiation of stem cells by activating the Notch signaling pathway, Osteoblast signaling pathway, and Wnt signaling pathway; modulate immune microenvironment by suppressing the MAPK signaling pathway, Toll-like receptor signaling pathway, and NF-kappa B signaling pathway; and promote angiogenesis by inducing the VEGF signaling pathway and the insulin pathway, thus apparently promoting bone regeneration in vivo (Fig. 13G). In another study, PVDF-based self-powered electrospun systems containing carboxylated MWCTNs have been engineered to create a mechanoresponsive “LOCK-ON/OFF” drug release platform (Fig. 13H) [276]. Carboxylated MWCNTs could adsorb weakly positively charged vancomycin (VAN) through electrostatic interactions because of their highly negative charged surface. Upon mechanical stimulation, the scaffold generates an electric field due to the piezoelectric effect, creating high-intensity positive and negative charge zones on either side of the carboxylated MWCNTs–VAN complex. The positive charge zone repelled the positively charged VAN molecules, while the negative charge zone attracted them to break away from the carrier, thus fulfilling the electrically stimulated responsive release of the drug for infected wound healing (Fig. 13I) [276].

Therefore, smart electroactive drug delivery devices achieve controlled electro-stimulated drug release in targeted areas via exogenous electric stimulation or self-powered electric signals, demonstrating great potential in tissue regeneration. In these therapeutic systems, electrical signals not only serve as therapeutic stimuli but also regulate drug release and even exert synergistic effects. Future research should advance large-scale animal experiments and long-term safety evaluations to facilitate the design of personalized treatment strategies.

## Conclusion and Prospects

The physiological cues of electric signals provide a fundamental rationale for scaffold design. Advances in electrospinning methods enable control over fiber architecture and scaffold composition, while material innovations ensure biocompatibility and tunable degradation. Strategies to confer electroactivity, including conductive polymers/nanomaterials and piezoelectric ceramics/polymers, effectively regulate critical regeneration processes such as cell proliferation, cell recruitment, and stem cell fate determination. Thus, EAESs that simulate ECM characteristics and possess potent electroactivity could overcome key limitations of electrotherapeutic devices, such as invasive electrodes and external power dependencies.

When electrospinning is used to fabricate EAESs for tissue regeneration, several critical factors require comprehensive consideration: (a) intrinsic electrophysiological characteristics of defective tissues; (b) ECM structure and properties for the rational selection of electrospinning methods and basic electrospinning materials; and (c) targeted electroactivity by the reasonable incorporation of conductive polymers/nanomaterials and piezoelectric ceramics/polymers. Despite promising potential for EAESs to treat defects in electroactive tissues, several certain limitations require further resolution. First, structural design complexities hinder functional integration. Conventional electrospinning typically yields dense 2D membranes with limited porosity, impeding critical cell infiltration and nutrient diffusion. Thus, electrospinning can be combined with other scaffold fabrication technologies such as 3D printing, gas foaming, and induced phase separation to fabricate advanced composites for tissue regeneration, which could well overcome these outstanding shortcomings. Second, the stability and long-term performance of electroactive polymers pose great challenges. Conducting polymers like PANI and PPy are prone to hydrolysis or oxidation in physiological environments, leading to diminished conductivity and reduced therapeutic efficacy over time. Piezoelectric polymers such as PVDF and PLLA exhibit low piezoelectric coefficients, which insufficiently generate robust electrical signals. Third, biocompatibility and safety concerns of electroactive materials remain paramount. Some electroactive particles such as CNTs, graphene, and BaTiO_3_ are correlated with poor biodegradability, and their long-term retention may cause chronic inflammation or fibrosis. In addition, adverse reactions like the generation of reactive oxygen species or localized pH shifts during electric stimulation may negatively impact tissue regeneration. Thus, it is highly needed to develop novel electroactive materials exhibiting excellent stability, desirable electrical properties, biodegradability, and biocompatibility.

Beyond advancements in scaffold fabrication and electroactive materials, challenges remain for the clinical translation of EAESs. Precise and safe electrical signal modulation presents a critical hurdle, because the lack of standardized stimulation parameters (intensity, frequency, and waveform), which vary greatly between tissues and often rely solely on animal data, heightens clinical translation risks. In addition, the difficulty in quantifying endogenous electrical signals generated in vivo by electroactive materials generally hinders further optimization of EAESs and explores the precise mechanism of action. Moreover, scaling production of EAESs faces substantial challenges due to persistent issues in maintaining batch-to-batch consistency and prohibitive sterilization costs.

Successfully overcoming these multifaceted limitations is critical for translating EAESs from promising preclinical platforms into viable clinical solutions, ultimately enabling them to serve as effective and potentially superior alternatives to current clinical electrotherapy devices. Their ability to provide spatially controlled, biomimetic electrical cues directly at the tissue interface holds substantial promise for achieving more targeted, efficient, and convenient therapeutic outcomes, paving the way for a transformative impact in regenerative medicine.
